# Asymmetric birth and death of type I and type II MADS-box gene subfamilies in the rubber tree facilitating laticifer development

**DOI:** 10.1371/journal.pone.0214335

**Published:** 2019-04-01

**Authors:** Anuwat Kumpeangkeaw, Deguan Tan, Lili Fu, Bingying Han, Xuepiao Sun, Xiaowen Hu, Zehong Ding, Jiaming Zhang

**Affiliations:** 1 International College, Huazhong Agricultural University, Lion Mountain, Wuhan, China; 2 Institute of Tropical Bioscience and Biotechnology, MOA Key Laboratory of Tropical Crops Biology and Genetic Resources, Hainan Bioenergy Center, CATAS, Haikou, Hainan Province, China; 3 Song Khla Rubber Research Centre, Department of Agriculture, Ministry of Agriculture and Cooperatives, Had Yai, Song Khla, Thailand; 4 Zhanjiang Experimental Station, CATAS, Zhanjiang, Guangdong Province, China; University of Naples Federico II, ITALY

## Abstract

The rubber tree (*Hevea brasiliensis* Muell. Arg.) is a rubber producing crop and contains specialized laticifers. MADS-box genes are a family of transcription factor genes that regulate plant development, especially floral organ and gametophyte development. 97 MADS-box genes were identified in the rubber tree through transcriptomes and genome mining. 93.8% of the genes were mapped onto the genome scaffolds in correspondence to the coverage (93.8%) of current version of sequenced genome. Phylogenetic analysis indicates that type II MADS-box genes have been more actively duplicated than their orthologous genes in Arabidopsis and rice, so that most (70, 72.2%) of the MADS-box genes in the rubber tree belong to type II subfamily. This is a high percentage compared to those in Arabidopsis (43.7%) and rice (56.8%). Moreover, 69 out of 70 type II genes in the rubber tree are transcribed, and they are mostly predominantly expressed in flowers, but some genes are predominantly expressed in laticifers, suggesting their roles in both flower and laticifer development. The number of type I genes in the rubber tree is only 27 (27.8%), a much smaller number compared to their orthologous genes in Arabidopsis (56.3%) and rice (43.2%). At the same time, most of the type I genes (55.6%, 15) in the rubber tree are silent and are probably pseudogenes. The high birth rate and low death rate of type II genes and low birth rate and high death rate of type I genes may corresponds to special developmental requirements in the rubber tree, e.g. the development of laticifer system for biosynthesis of cis-polyisoprene, the rubber. Moreover, atypical MIKC* factors (e.g. *HbMADS1* in S-clade, and *HbMADS20* in P-clade) are identified. These genes are diverged to typical MIKC* genes in sequences and facilitate functions required in laticifer development and rubber biosynthesis, which is not necessary in Arabidopsis and rice.

## Introduction

MADS-box genes have long evolutionary history and spread widely in plants, animals, and fungi. They were found independently in different organisms and were once named as MINICHROMOSOME MAINTENANCE1 (MCM1) in *Saccharomyces cerevisiae* [1], AGAMOUS in *Arabidopsis thaliana* [2], DEFICIENS in *Antirrhinum majus* [3], and SERUM RESPONSE FACTOR (SRF) in *Homo sapiens* [4]. All these genes encode a highly conserved N-terminal DNA binding domain of 55–60 amino acids in length and were given the acronym MADS domain using the first letters of the above genes [5]. The MADS domain may have ancient origin. Homology searches in the microbial database suggested that the MADS domain originated from the DNA binding subunit A of topoisomerases IIA, and gave rise to SRF-like and MEF2-like MADS-box genes after gene duplication that occurred in extant eukaryotes [6].

Based on sequence conservation in the MADS domain, the MADS-box transcription factors are classified into type I (SRF-like) and type II (MEF2-like) [7]. Animal type I and II genes can be separated into monophyletic groups. Plant type I genes do not group into a monophyletic cluster [8] and have undergone a faster rate of birth and death during evolution compared with animal type I (SRF) and plant type II (MIKC) genes; therefore, early reports concluded that plant type I genes were probably of minor functional importance in comparison to type II genes [9, 10]. Arabidopsis type I genes were divided into three subfamilies Mα, Mβ, and Mγ, although AGAMOUS LIKE33 was not assigned to any of these [11], while type II MADS-box genes were divided into two main subfamilies MIKC^c^ and MIKC* [8].

Plant MADS-box transcription factor genes are involved in the regulation of a wide range of plant biological processes including plant development and stress responses. Their involvement in flower development has been extensively studied, and mutations in the MADS-box genes that control flower development resulted in phenotypic variations of flowers in Arabidopsis [12, 13]. For example, stamens were transformed into petals in the mutant agamous-1, sepals were transformed into leaves in the mutant apetala2-1, petals were transformed into sepals and stamens were transformed into carpels in the mutant apetala3-1 [12]. In addition, double mutations ap2-1 and pi-1 resulted in loss of organs [12]. Similar phenomenon in flower regulation was also observed in other plant species [3, 14, 15]. MADS-box genes are also involved in cell proliferation and differentiation modulation during vegetative development [16, 17], for example, cell cycle [18], cell differentiation [16], and cell degeneration [19]. The roles of MADS-box genes in stress-responses were also reported [20–22].

The rubber tree (*Hevea brasiliensis* (Willd.) Muell.-Arg) is a member of the Euphorbiaceae family, and is the major producer of natural rubber. There are approximately 2,500 plant species that synthesize rubber, however, the rubber tree accounts for approximately 98% of natural rubber worldwide [23, 24]. Natural rubber consists of primarily cis-1,4-polyisoprene [25] and is synthesized in specialized laticifer cells. There are primary and secondary laticifers and they are morphologically and functionally diversified [26]. Little is known about the role of MADS-box gene family in the development of laticifer cells in the rubber tree. Four MADS-box genes, designated as *HbMADS1*, *HbMADS2*, *HbMADS3*, and *HbMADS4*, have been isolated from the rubber tree, and all are highly transcribed in the laticifer cells [27, 28]. The transcription of *HbMADS1*, *HbMADS3*, and *HbMADS4* was enhanced in the laticifers by jasmonate, while *HbMADS2* was not induced. Moreover, transient over-expression of *HbMADS4* in transgenic tobacco plants significantly suppressed the expression of *HbSRPP*, a key gene involved in rubber biosynthesis, suggesting that *HbMADS4* is a negative regulator of *HbSRPP* [28].

Currently, four genome accessions and multiple transcriptomes of the rubber tree have been released. This paper reports our genome-wide analysis of the MADS-box gene family and their regulatory function in the rubber tree.

## Materials and methods

### Plant materials

The rubber tree variety Reyan7-33-97 was grown in the experimental farm of Chinese Academy of Tropical Agricultural Sciences located in Danzhou, Hainan Province, China. The trees used for sample collection had been grown for 10 years. Flower, root, bark, leaf, and latex were collected at eight in the morning in July, 2017. The samples were frozen immediately in liquid nitrogen and stored in -80°C until use.

### Transcriptome assembly

The Illumina RNA-seq reads (LVXX01000000) of the transcriptomes in different tissues of the rubber tree variety Reyan7-33-97, including root, leaf, bark, male flower, female flower were downloaded in NCBI database. The transcriptome RNA-seq reads of the primary and secondary laticifers were from the previous research [26] and were deposited in NCBI database under accession number SRP095295. The RNA-seq reads were de novo assembled using Trinity Version 2.1.1 [29], according to the recommended protocol. In addition, bowtie 2 [30] was used as a plug-in program in the Trinity software.

### Gene identification and nomenclature

A total of four MADS-box proteins of the rubber tree were obtained by name searches in the NCBI database. To performed genome-wide mining, the whole genome sequences of variety Reyan7-33-97 were obtained from Professor Caorong Tang, Institute of Rubber Research, Chinese Academy of Tropical Agricultural Sciences [31], which were also downloadable from GenBank using accession number SRP069104. Local tBLASTn searches were performed using protein sequences of MADS-box genes from Arabidopsis and rice [32] as queries against the rubber tree genome and transcriptome databases. The candidate transcripts were translated into peptide sequences using TransDecoder v5.2.0 (http://transdecoder.github.io) using known MADS-box proteins as blast databases. The correct transcripts were mapped to the genome sequences of the rubber tree variety Reyan7-33-97. The transcripts that were mapped to the same location of the same scaffold were considered as the same gene. After removing redundant sequences, each MADS-box candidate was further verified using the Web CD-Search Tool (http://www.ncbi.nlm.nih.gov/Structure/bwrpsb/bwrpsb.cgi) [33], the E-value cutoff was 0.01. The verified genes were named using the prefix *HbMADS* and a number in order of discovery. The molecular weight and isoelectric point of each MADS-box protein were calculated using the IPC [34].

### Phylogenetic and sequence analysis

Protein sequences were aligned using ClustalX [35]. Phylogenetic tree was conducted by MEGA7 [36] using Neighbor Joining (NJ), Maximum Likelihood (ML), and Minimum Evolution (ME) methods. Bootstrap analysis was performed using 1000 replicates with the pair-wise gap deletion mode. The exon-intron structure was analyzed by aligning the CDS and the relevant genomic sequence with MacVector version 13.5 (MacVector, Inc., Apex, USA). The exon-intron graph was prepared with GSDS2.0 [37].

Protein motifs were predicted by MEME suite with optimum motif width ranged from 10 to 60 and maximum number of motifs equal to 30 and cutoff threshold equal to e-8. The predicted motifs of MADS-box factors were annotated by searching against InterPro database (http://www.ebi.ac.uk/Tools/pfa/iprscan/).

### RNA isolation and qRT-PCR analysis

Total RNA was extracted using RNA plant reagent kits (Tiangen Company, Beijing, China). Differential expression of *HbMADS* genes in root, leaf, bark, flowers, and the primary and secondary laticifers was examined by qRT-PCR method using SYBR-green (TaKaRa Biotechnology Co. Ltd, Dalian, China) and Stratagene Mx3005P system (Stratagene, CA, USA) as previously described [38], three replicates were performed. The eukaryotic translation initiation factor (eIF1Aa) (HQ268022) [39] was used as an internal control. Gene specific primers were designed using Primer 5.0 method incorporated in MacVector (S1 Table). The specificity of each primer pair was confirmed as previously described [26]. The relative mRNA abundance was calculated by 2^-ΔΔCt^. The significance of difference was analyzed by one-way ANOVA at the 5% significance level using SPSS Statistics 24.0 (IBM, USA).

## Results

### Identification and classification of MADS-box genes in the rubber tree

To identify MADS-box genes in the transcriptomes, the RNA-seq reads of the transcriptomes in leaf, bark, root, male flower, female flower, seeds, and primary and secondary laticifers of the rubber tree variety Reyan7-33-97 were de novo assembled tissue by tissue and/or as a whole plant. The statistics of the assembled transcriptomes is provided in S2 Table. The largest numbers of Trinity ‘genes’ and transcripts were obtained by using the clean reads from all tissues as a whole plant, which are 388,766 and 158,136 for Trinity transcripts and Trinity ‘genes’, respectively. The lowest numbers of Trinity ‘genes’ and transcripts were obtained in the secondary laticifer, which are only 50,504 and 28,726 for transcripts and ‘genes’, respectively, accounting for less than 20% of Trinity ‘genes’ in the whole plants. The ‘gene’ number in the primary laticifer is also small, only 36,944. Trinity ‘gene’ numbers in all other tissues are above 64,000 (S2 Table). These results are reasonable, since either the primary or the secondary laticifers belong to a single cell type, other tissues are all mixtures of many cell types. Different cell types may recruit different sets of genes for their biological functions, resulting in larger number of total genes in a tissue with many cell types. It is interesting that the total GC content of the transcriptomes in the primary and the secondary laticifers are the highest (>42%,) among the tested tissues, suggesting that the gene set recruited by laticifer cells has higher GC content.

A total of 605 transcripts and 91 genomic genes were obtained as MADS-box gene candidates by tBlastn searches in the transcriptomes and genome databases, respectively. Redundant transcripts that arose from alternative splicing and/or highly similar sequences that were mapped to the same chromosome locations [31] were removed. The longest transcripts containing all exons are kept as representative transcripts of the genes. A total of 97 non-redundant MADS-box genes were identified, including four genes (*HbMADS1* to *HbMADS4*) that have been described [27, 28] and 16 genes that were solely deduced from genome sequences. The newly identified genes were named from *HbMADS5* to *HbMADS97* according to the order of identification and deposited in the GenBank database under accession numbers MG922673-MG922769 (S3 Table). They encode proteins of 152 to 383 amino acid residues in length with an average length of 243 aa and 27.8 KDa in molecular weight. Their pI ranged from 5.1 to 11.2. All the genes were verified to be MADS-box genes in the CDD and Pfam databases.

**Fig 1:**
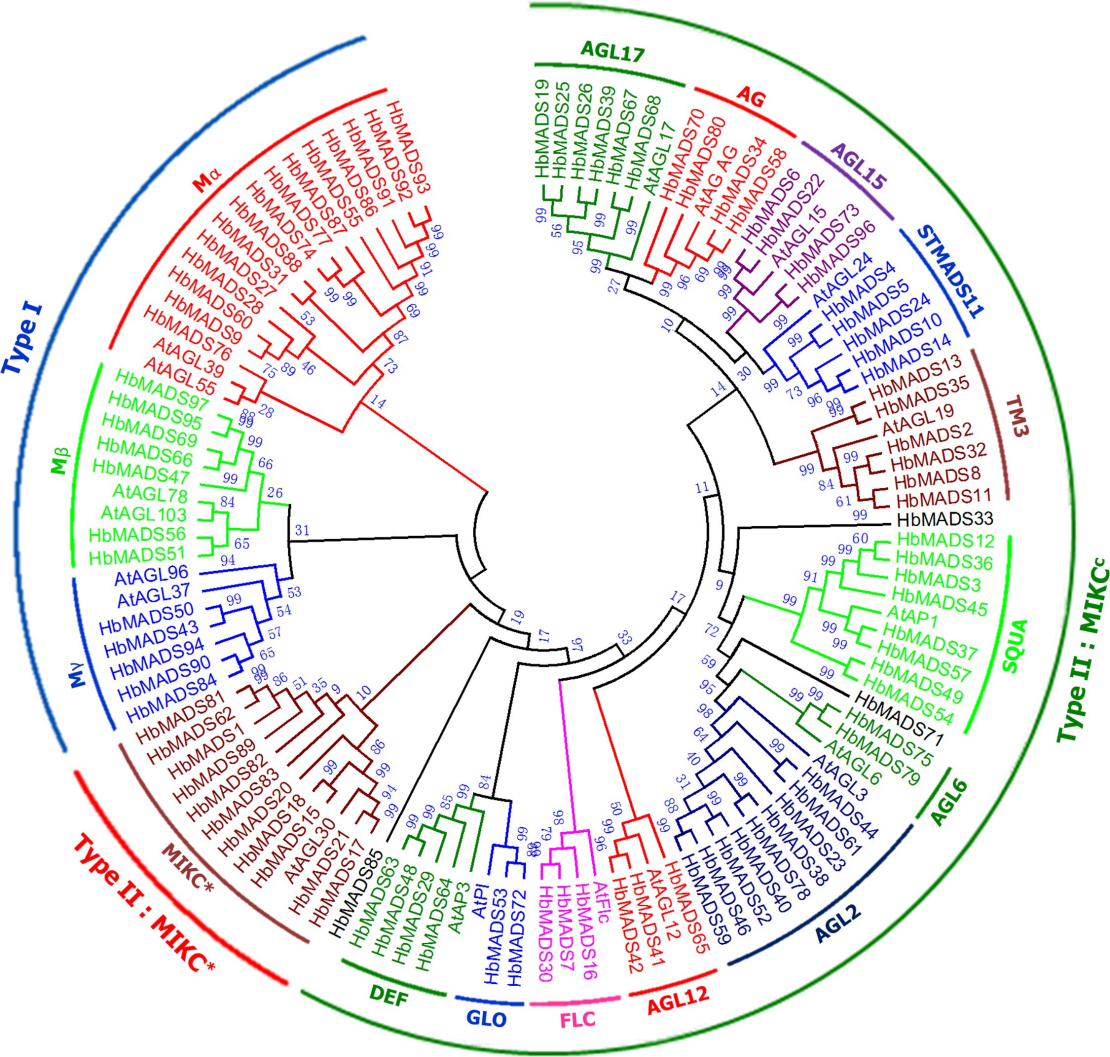
Maximum likelihood tree of MADS-box factors in the rubber tree. A total of 134 MADS-box proteins including 97 from the rubber tree (prefixed with ‘Hb’) and 19 representative sequences of well known clades in Arabidopsis (prefixed with ‘At’) were aligned using ClustalX2.0 [35]. The evolutionary distances were computed using the number of differences method [40]. All positions with less than 80% site coverage were eliminated, and a total of 256 positions in the final dataset were used to create the tree. The evolutionary analyses were conducted in MEGA7 [36]. The accession numbers of the Arabidopsis MADS-box proteins are provided in S4 Table.

The phylogenetic relationships of the HbMADS proteins were analyzed by using representative MADS proteins from Arabidopsis as references [11]. All HbMADS are clustered into five groups, including Mα (15), Mβ (7), Mγ (5), MIKC* (11), and MIKC^c^ (58) with the number of genes in brackets, except HbMADS85, which is clustered in the type II subfamily, but is not clustered into any of the two type II subgroups MIKC^c^ and MIKC* (Fig 1). HbMADS85 has the shortest peptide (152 aa) in HbMADS family of the rubber tree. Further phylogenetic analysis using all MADS-box proteins from Arabidopsis and rice clustered HbMADS85 into MIKC* clade (S1 Fig). Therefore, the final MIKC*-type gene number in the rubber tree is 12.

### Gene structure and conserved motif analysis

Out of the 97 MADS-box genes in the rubber tree, 91 genes (93.8%) are mapped on the scaffolds of the rubber tree variety Reyan7-33-97 (S3 Table) in correspondence to the 93.8% coverage of the sequenced genome [31]. Only six members (*HbMADS12*, -*23*, -*29*, -*36*, -*71*, and -*76*) are not mapped on the genome sequences. One member gene *HbMADS16* over-span two scaffolds (Scaffold1599 and -1612) with a gap of approximately 2500 bases in intron 2 as revealed by aligning with a homologous scaffold (BDHL01019014) identified in the genome sequence of another variety BPM24 [41].

**Fig 2:**
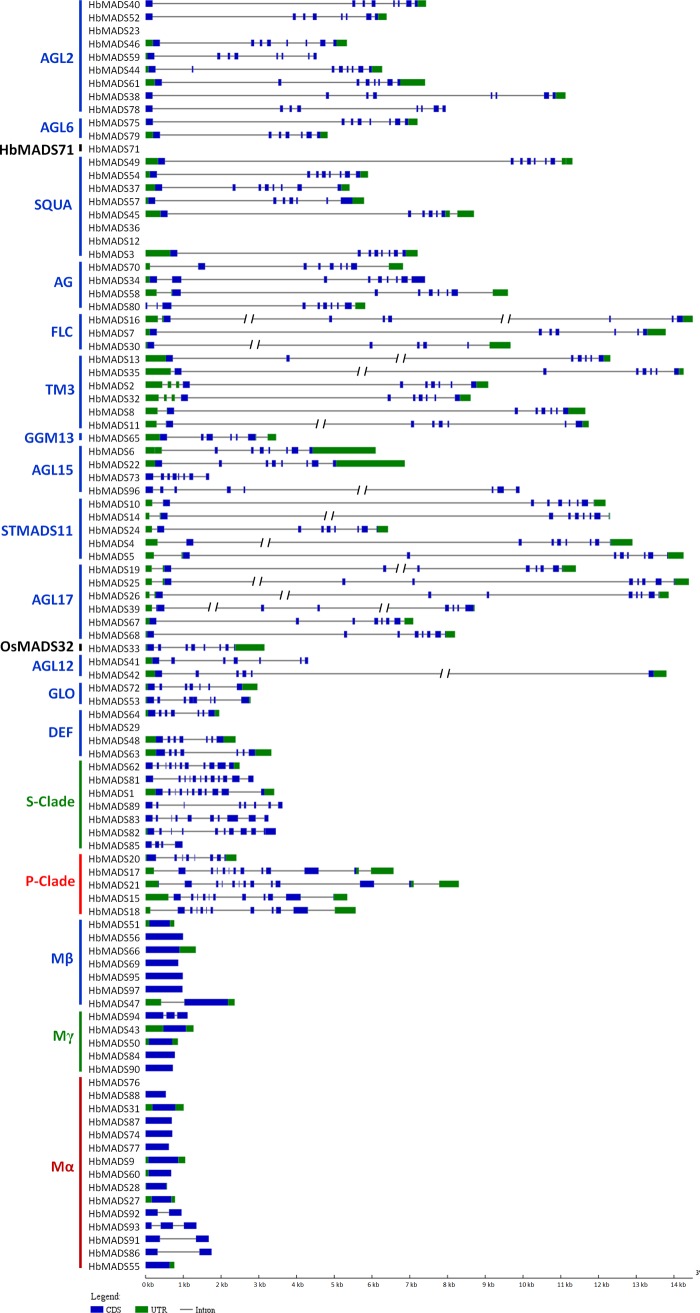
Gene structures of MADS-box transcription factors in the rubber tree. The graph was prepared with GSDS2.0 [37]. The slashes in introns indicate positions of cutouts to make the sequences fit in page. The lengths of cutouts are respectively 30 kb in intron 2 and 12 kb in intron 5 of *HbMADS16*, 5 kb in intron 2 of *HbMADS14*, 10 kb in intron 2 of *HbMADS11*, 5 kb in intron 2 of *HbMADS13*, 10 kb in intron 3 of *HbMADS19*, 10 kb in intron 2 and 4 kb in intron 3 of *HbMADS25*, 21 kb in intron 2 of *HbMADS26*, 10 kb in intron 1 of *HbMADS30*, 5 kb in intron 2 of *HbMADS35*, 14 kb in intron 2 and 3 kb in intron 9 of *HbMADS39*, 10 kb in intron 2 of *HbMADS4*, 1 kb in intron 5 of *HbMADS42*, 22 kb in intron 1 of *HbMADS7*, and 5 kb in intron 5 of *HbMADS96*.

Gene structure of *HbMADS* genes was analyzed individually by aligning the transcripts with their genome sequences (Fig 2). Overall, type II genes are longer and contain more exons than type I genes. The length of type I genes ranges from 537 to 2,361 bp with an average of 1,006 bp and contains 1–3 exons, while the length of type II genes ranges from 887 to 56,497 bp with an average length of 10,270 bp, which is ten times the size of the average length of type I (S3 Table). *HbMADS16* has the largest gene size, while *HbMADS85* has the shortest gene size. Type II genes also have more exons ranging from 7 to 13 compared to type I genes, except for *HbMADS85*, which has only four exons due to deletion mutation of six exons (exon 2, 3, 4, 7, 9, and 10, S2 Fig). *HbMADS85* is therefore probably a pseudogene, although it is transcribed in male flowers (see below).

**Fig 3:**
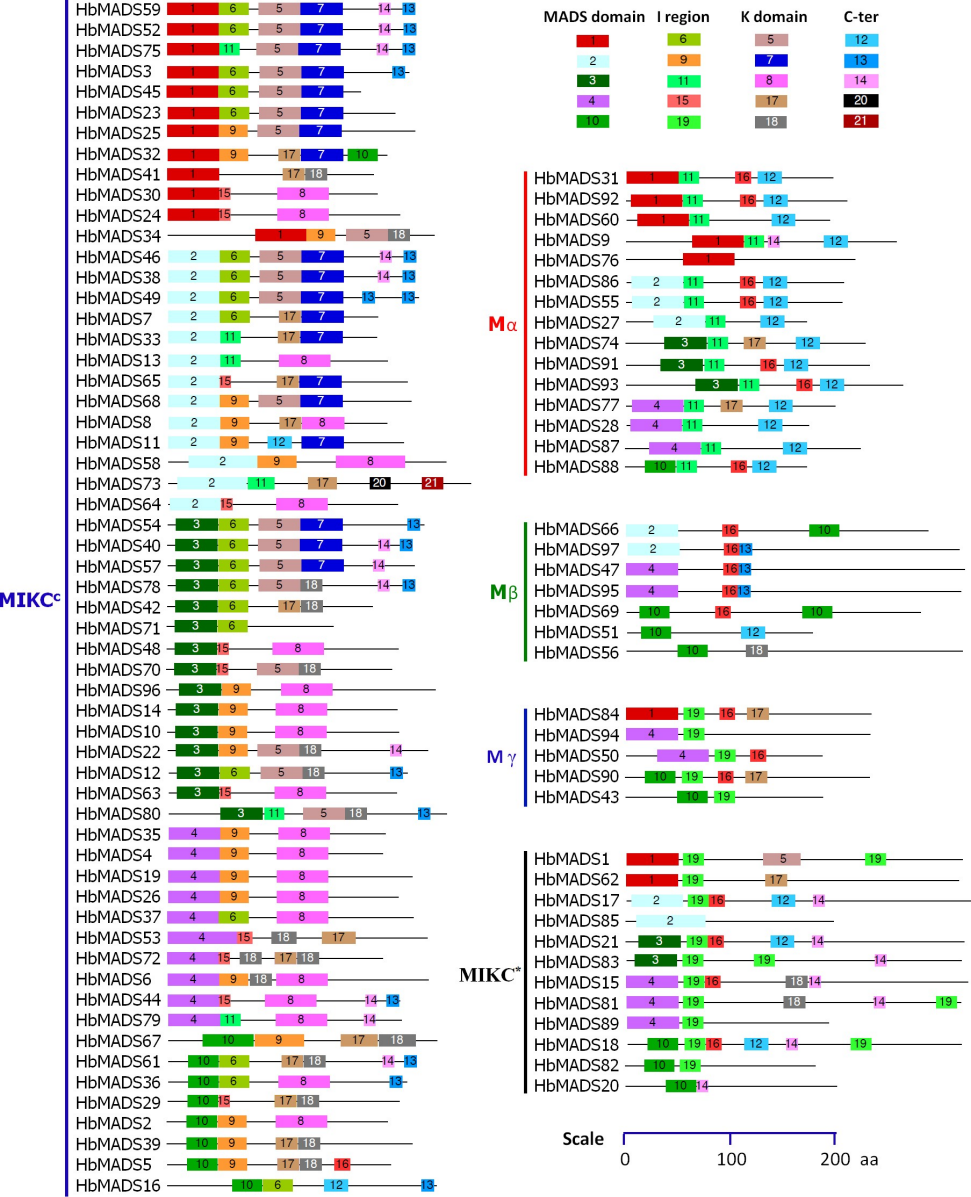
Conserved motifs of MADS-box factors in the rubber tree. The motifs were identified with MEME Suite 5.03 [42] and confirmed in InterPro database (http://www.ebi.ac.uk/interpro/). Motifs are shown by different color boxes, while non-conserved sequences are shown in grey line. Length of motifs is present in scale.

In general, closely related genes in phylogenetic analysis shared similar exon-intron structures. For example, AGL12-like, DEF-like, and GLO-like MIKC^c^ genes contain 7 exons; AGL2-like and AGL15-like MIKC^c^ genes contain 8 exons, STMADS11-like MIKC^c^ genes contains 9 exons. Most S-clade MIKC* genes contain 10–12 exons, while most P-clade MIKC* genes contain 12–13 exons, except for *HbMADS20*, which contains only eight exons. *HbMADS20* is diverged compared to other P-clade MIKC* genes not only in gene structure, but also in protein motif composition (Fig 3), as well as expression pattern (see below). This gene is specifically expressed in the secondary laticifer cells, suggesting that this gene has been recruited for regulation of laticifer development and rubber biosynthesis after its mutation or vice-versa.

In total, 21 motifs were identified by using MEME methods [42] and subsequently annotated with InterPro database. The classical MIKC-type (MIKC^c^) MADS proteins have typical domains of type II MADS-box proteins, including a MADS domain, an intervening (I) domain, a keratin-like coiled-coil (K) domain, and a C-terminal (C) domain (Fig 3). The MIKC*-type proteins have also typical MADS domain of type II MADS protein, however, they do not have classical K domain, except HbMADS1. The type I MADS all have typical MADS domains and a diversified C-terminal domain (Fig 3).

### The MIKC^c^-type MADS-box genes are duplicated actively in subgroup-specific pattern and play both conserved and diverged functions

MIKC^c^-type MADS subfamily is composed of 58 members, accounting for 59.8% of the MADS-box factors in the rubber tree, which is a larger percentage compared to Arabidopsis (37.5%) and rice (50.7%). This phenomenon indicates that MIKC^c^-type MADS genes have been duplicated more actively than other subfamily members in the evolution history of the rubber tree.

**Fig 4:**
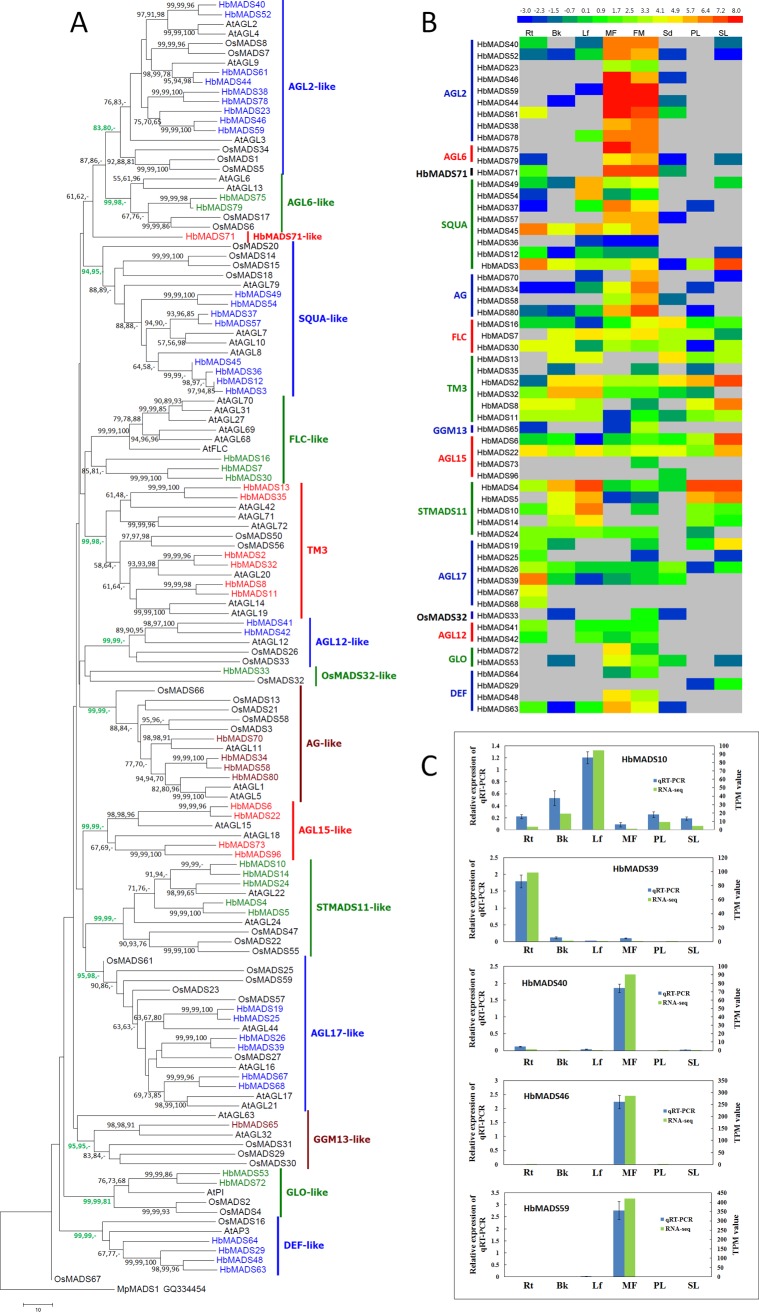
Phylogenetic relationships and expression profile of MIKC^c^-type MADS-box proteins in the rubber tree. A, a Neighbor Joining Tree. The evolutionary history was inferred using Neighbor Joining (NJ), Maximum Likelihood (ML) and Minimum Evolution (ME) methods, respectively. A total of 133 MIKC^c^ factor sequences from the rubber tree (prefixed with ‘Hb’), Arabidopsis (prefixed with ‘At’), and rice (prefixed with ‘Os’) are involved in the analysis. The tree is rooted with an ancient MADS-box protein MpMADS1 (GQ334454) from a liverwort species *Marchantia polymorpha*. All positions with less than 80% site coverage were eliminated and 177 positions were used in the final datasets to create the tree. The analysis were conducted in MEGA7 [36]. B, expression profiles of MIKC^c^-type MADS factor genes in the rubber tree. C, Real-time quantitative PCR analysis in comparison to RNA-seq results. Rt, root; Bk, bark; Lf, leaf; MF, male flower; FF, female flower; PL, primary laticifer; SL, secondary laticifer. The accession numbers of MADS-box proteins from Arabidopsis and rice are provided in S4 and S5 Tables.

Phylogenetic analysis of all MIKC^c^ members in the rubber tree, Arabidopsis and rice revealed that the MIKC^c^-type proteins are clustered into 13 main clades (Fig 4A), except for HbMADS71 and HbMADS33 from the rubber tree and OsMADS32 and OsMADS67 from rice, which cannot be well clustered into any clades. OsMADS32 in rice has been recognized as an independent subgroup by itself [20]. HbMADS33 is clustered together with OsMADS32, and thus designated as an OsMADS32-like MADS factor, however, the bootstrap support is not strong (Fig 4A). Arabidopsis does not have a member in the OsMADS32-like clade. HbMADS71 is an independent taxon in all analysis, which is also supported by Fig 1. Therefore, HbMADS71 is designated as a HbMADS71-like MADS factor, which represents the most diverged member in the MIKC^c^-type MADS factors. As for OsMADS67 in rice, it contains a peptide of only 57 aa, and is probably a pseudogene.

Out of the 13 main subgroups, 11 subgroups contain MADS factors from all three species, indicating that these subgroups occurred before separating of monocots and dicots (Fig 4), in agreement with the hypothesis that there were 11 seed plant-specific clades in the MRCA of extant seed plants [43]. Rice does not have any members in the FLC-like and AGL15-like clades.

Gene numbers of the rubber tree in seven clades, including AGL2-, SQUA-, AGL15-, STMADS11-, AGL12-, GLO-, and DEF-like MADS are twice as many as those in Arabidopsis and rice (Fig 4A), which is the main cause for the rubber tree to have a larger MIKC^c^ subfamily than Arabidopsis and rice.

Interestingly, HbMADS71 outgroups AGL2-like and AGL6-like clades (Fig 4A), suggesting that HbMADS71 may represent the common ancestor of the AGL2- and AGL6-like MADS-box factors. Transcriptome analysis results show that HbMADS71 is predominantly expressed in male and female flowers (Fig 4B and 4C), suggesting that this primitive protein is still functioning. Surprisingly, all the AGL2-like and AGL6-like MADS factors are also dominantly expressed in male and female flowers, indicating that the expression pattern and biological function of these two clade members are highly conserved and can be traced back to the occurrence of the common ancestor of the monocots and dicots.

Besides AGL2- and AGL6-like subgroups, the AG-, GLO-, GGM3-, and DEF-like *HbMADS* genes are also predominantly expressed in flowers, while AGL17-like *HbMADS* genes are predominantly expressed in root. Most genes in the AGL15-, STMADS11-, and TM3-like MIKC^c^ subgroups are highly expressed in the primary and/or secondary laticifers, however, since laticifers exist in all other tissues, these genes are also expressed in other tissues although at relatively low levels due to RNA dilutions by other cell types. The expression pattern in the SQUA-like MIKC^c^ clade is the most divergent, which includes genes (*HbMADS49* and *-54*) that are predominantly expressed in leaf and genes (*HbMADS37* and *-57*) that are predominantly expressed in flowers, and also genes (*HbMADS3* and *-45*) that are predominantly expressed in root and other tissues. Interestingly, the genes with similar expression patterns are phylogenetically clustered together (Fig 4A), indicating that their expression pattern are still conserved after duplication. In total, 11 genes including the previously reported *HbMADS2*, *-3*, and *-4* [27] are highly expressed in laticifers indicating that MIKC^c^-type MADS are involved in laticifer development and rubber biosynthesis.

Real-time qRT-PCR was performed to verify the RNA-seq results. Results show that all the tested genes (*HbMADS10*, *-39*, *-40*, *-46*, *-59*) have similar expression patterns as the RNA-seq results (Fig 4C).

### The MIKC*-type genes are highly duplicated in rubber tree and may have evolved both conserved and diverged biological functions

**Fig 5:**
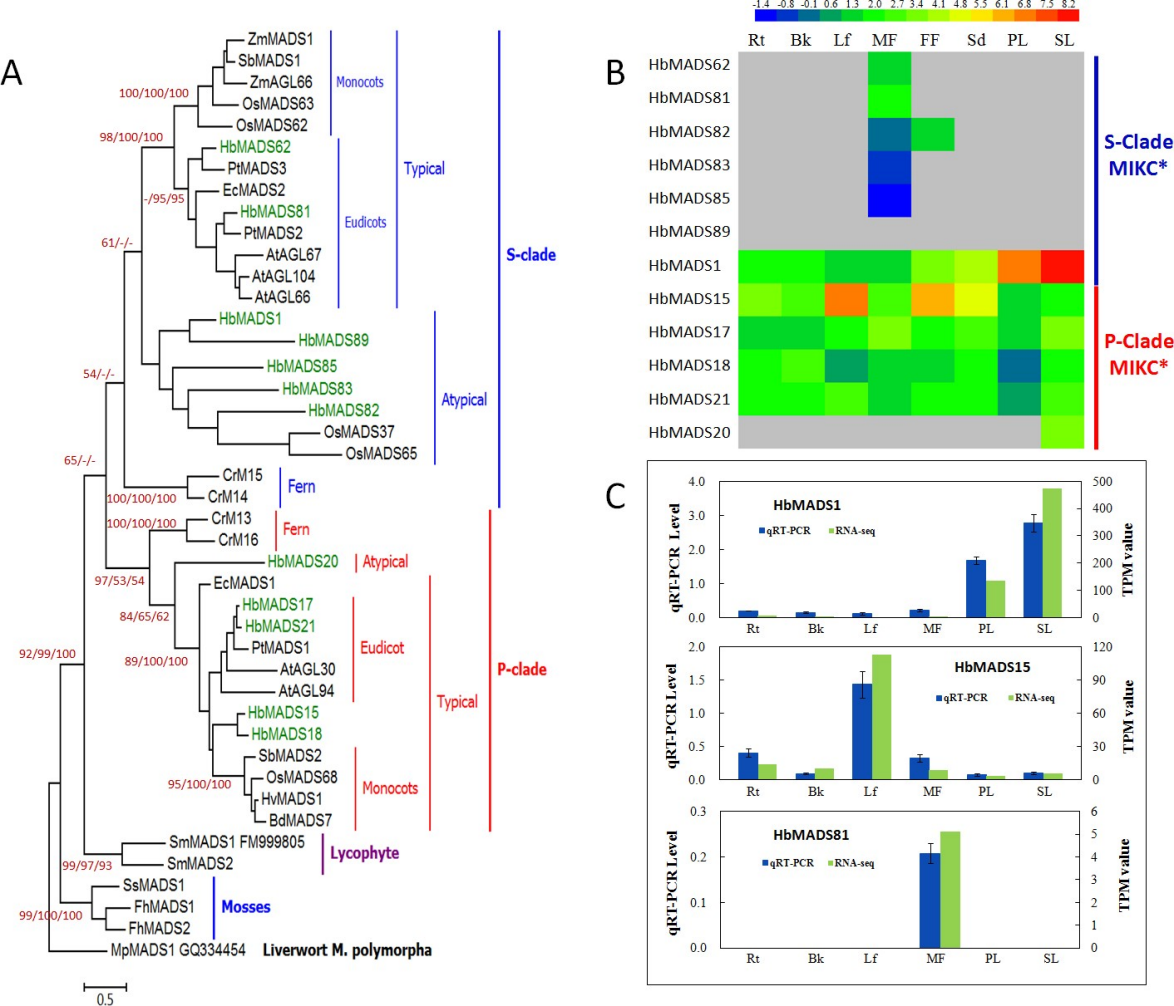
Phylogenetic relationships and expression profiles of MIKC*-type MADS-box proteins in the rubber tree. A, a Maximum likelihood tree. The evolutionary history was inferred using NJ, ML, and ME methods. A total of 42 MIKC* MADS-box proteins from rubber tree (prefixed with ‘Hb’), Arabidopsis (prefixed with ‘At’), rice (prefixed with ‘Os’), *Zea mays* (prefixed with ‘Zm’), *Ceratopteris richardii* (prefixed with ‘Cr’), *Eschscholzia californica* (prefixed with ‘Ec’), *Funaria hygrometrica* (prefixed with ‘Fh’), *Sphagnum subsecundum* (prefixed with ‘Ss’), *Populus trichocarpa* (prefixed with ‘Pt’), *Hordeum vulgare* (prefixed with ‘Hv’), *Brachypodium distachyon* (prefixed with ‘Bd’), and *Selaginella moellendorffii* (prefixed with ‘Sm’) are involved in the analysis. The accession numbers of the reference sequences are provided in S6 Table. The tree is rooted with an ancient MADS-box protein MpMADS1 (GQ334454) from a liverwort species *Marchantia polymorpha*. All positions with less than 80% site coverage were eliminated and a total of 390 positions were used in the final dataset. Bootstrap values of conserved clades as calculated using ML, NJ, and ME methods are provided above or below the branches. Scale bar represent 0.5 substitutions. B, MIKC* expression profile by RNA-seq. **C,** real-time RT-PCR results of representative genes. Rt, root; Bk, bark; Lf, leaf; MF, male flower; FF, female flower; PL, primary laticifer; SL, secondary laticifer.

Similar to the MIKC^c^-type MADS-box factors, the MIKC*-type genes in the rubber tree are also duplicated in a more active pattern as compared to the orthologous genes in Arabidopsis and rice. A total of 12 MIKC* genes were identified, which is a much larger number than those in Arabidopsis (5) and rice (5). Phylogenetic analysis indicates that there are both conserved and diverged MIKC* genes in the rubber tree compared to Arabidopsis and rice. Seven members (*HbMADS1*, *-62*, *-81*, *-82*, *-83*, *-85*, *-89*) are clustered in the S-clade and five (*HbMADS15*, *-17*, *-18*, *-20*, and *-21*) in the P-clade (Fig 5A). In previous studies in rice, although five were grouped in MIKC*-type MADS[20], only three of them, OsMADS62, -63, and -68 were identified as genuine MIKC*-type proteins [44, 45]. The rest two (OsMADS37 and -65) were regarded as not genuine MIKC* and omitted from analysis [46]. We included these two proteins in our analysis and revealed that OsMADS37 and -65 together with four HbMADS (HbMADS1, -82, -83, -85, and -89) are clustered as an atypical S-clade (Fig 5A). The typical S-clade proteins include those from both monocots and eudicots as previously reported [45, 46]. Similarly, the typical P-clade proteins also include members from both monocots and eudicots including four HbMADS (HbMADS15, -17, -18, and -21) from the rubber tree, and they are outgrouped by an atypical member (HbMADS20), suggesting that HbMADS20 may represent a common ancestor of the P-clade MIKC*-type MADS-box proteins of monocots and dicots.

The typical S-clade and P-clade MIKC* members in rice and Arabidopsis have conserved expression pattern. Rice contains two in the typical S-Clade (OsMADS62 and -63) and one in the typical P-Clade (OsMADS68), they are all specifically expressed in flowers and involved in pollen development and pollen tube growth [46]. All the five Arabidopsis MIKC* are clustered in either the typical S-clade or P-clade in our analysis (Fig 5A), and they all, except AGL67, are almost exclusively expressed during pollen development [10, 47, 48]. It is interesting that the two typical S-clade members in the rubber tree (HbMADS62 and -81) are also specifically expressed in male flowers (Fig 5B), suggesting their conserved function in pollen development. Three atypical S-clade members, HbMADS82, -83, and -85 are also specifically expressed in flowers, especially in male flowers, suggesting that S-clade MIKC* have been involved in flower development before the separation of monocots and dicots.

However, the atypical S-clade MIKC* gene, *HbMADS1* represents the most highly transcribed MIKC* genes in the rubber tree, but is predominantly expressed in the secondary laticifer, followed by the primary laticifer, seeds, and other tissues (Fig 5B and 5C). *HbMADS1* is the first characterized MADS-box gene in the rubber tree and was reported to be expressed at different levels with the highest transcription in latex [27]. It is worth mentioning that there are laticifers in all the tested tissues including leaf, bark, flowers and seeds, therefore, our result together with previous research do not rule out that *HbMADS1* is specifically expressed in the laticifer cells. These results suggest that the atypical S-clade MIKC* genes, such as *HbMADS1*, may have evolved biological functions different to its orthologous genes in Arabidopsis and rice, it may represent the most functionally diverged MIKC*-type MADS-box gene.

The number of P-clade MIKC* genes in the rubber tree are highly duplicated to five in comparison to two in Arabidopsis and only one in rice. The expression patterns of the typical P-clade members are more or less similar within the rubber tree, but are different compared to their orthologous genes in Arabidopsis and rice. *HbMADS15* is highly expressed in leaf and female flowers and relatively weak expressed in other tissues (Fig 5B). The rest typical P-clade members (*HbMADS17*, *-18*, and *-21*) are almost evenly expressed in all tissues at low levels. However, the atypical P-clade member *HbMADS20* is specifically expressed in the secondary laticifer cells. These results suggest that the P-clade MIKC* genes in the rubber tree may play more complex roles than their orthologous genes in Arabidopsis and rice.

Taken together, the typical MIKC*-type MADS factors are conserved and mainly play roles in flower and pollen development, although some typical P-clade proteins also play roles in the regulation of vegetative growth. In contrast, the atypical MIKC* factors (e.g. HbMADS1 in S-clade, and HbMADS20 in P-clade) are more diverged and facilitate functions required in laticifer development and rubber biosynthesis, which is not necessary in Arabidopsis and rice.

### Type I MADS-box transcription factor genes in the rubber tree are restricted in duplication and are mostly silent

Quite in contrast to the active duplication of the type II MADS-box transcription factor genes, the number of type I MADS factors in the rubber tree are strongly limited compared to that in Arabidopsis and rice. The rubber tree has a genome size of 1.27 Gb, which is 10 times the size of Arabidopsis [31], but its type I factor gene number is only 27, just 45% of the type I factor gene number (61) in Arabidopsis. Rice (32) also has more type I MADS genes than the rubber tree. The driving force for this asymmetric duplication of the MADS-box transcription factor gene family may come from special requirements for the development of the rubber tree, e.g. development of laticifer system, which does not exist in Arabidopsis and rice.

**Fig 6:**
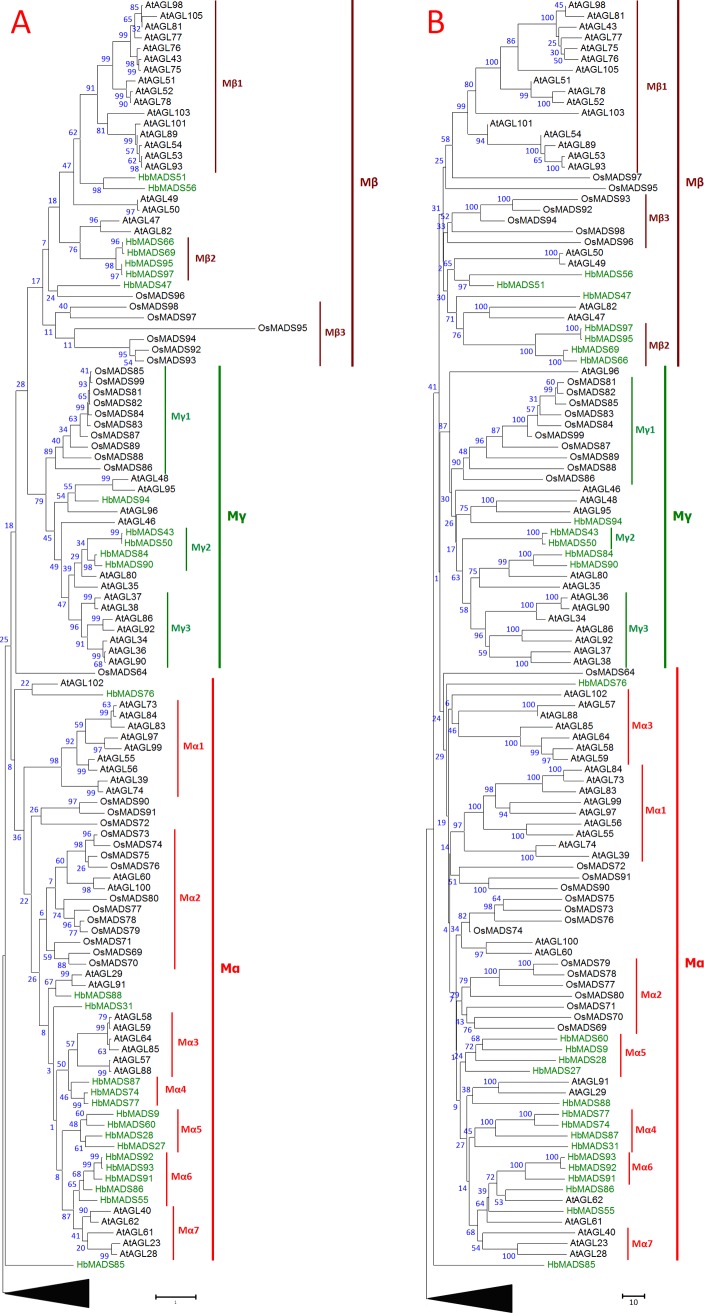
**Maximum Likelihood (A) and Neighbor Joining (B) trees of type I MADS-box transcription factor sequences.** A total of 127 amino acid sequences were used in the analysis, including all type I MADS from the rubber tree (prefixed with ‘Hb’), Arabidopsis (prefixed with ‘At’), and rice (prefixed with ‘Os’), and one type II (HbMADS85) from rubber tree, and outgroups from distinct species (CAX33869, CAX33874, GQ334454, XP_024027649, XP_021284791, PAN24898, AST36051, CAX33873, and CAX33871). The HbMADS85 is used to confirm that it does not belong to type I subfamily because it is not classified into either MIKC^c^ or MIKC* in Fig 1. The outgroups are represented with a black triangle to simplify the tree. All positions with less than 80% site coverage were eliminated and 153 positions were used in the final datasets to create the trees. The analysis were conducted in MEGA7 [36].

Phylogenetic analysis using the type I MADS-box factors from Arabidopsis and rice as reference sequences revealed that all type I MADS factors from the rubber tree are clustered into Mα, Mβ, and Mγ subfamilies by ML, NJ, and ME methods. However, the bootstrap supports for all subfamilies are low, although Mγ clade has 79%, 87%, and 87% bootstrap probabilities in the trees built with ML, NJ, and ME methods, respectively (Fig 6). These results indicate faster evolution of type I MADS as compared to type II MADS, in agreement with previous investigations, in which type I MADS have experienced rapid duplications and loss of function during species formation [8]. However, the driving force controlling the rapid duplication of type I MADS genes is apparently weaker in the rubber tree than in Arabidopsis and rice resulting in much smaller number of type I members in the rubber tree.

Most of the type I gene duplications occurred after the separation of the rubber tree, Arabidopsis, and rice, which resulted in species-specific subclades. Some subclades are numerically designated in Fig 6 for convenience of description. The largest species-specific Mβ subclade for Arabidopsis is Mβ1 with 16 members. Rice also has a six-member subclade (Mβ3) in the Mβ group. Four out of the seven Mβ factors in the rubber tree are clustered in the Mβ2 subclade. Mγ group shows even bigger difference among species. The rubber tree has only five Mγ factors and four are clustered in Mγ2 subclade, while Arabidopsis and rice have respectively 16 and 10 and they are mostly clustered in subclades Mγ1 and Mγ3 for rice and Arabidopsis, respectively (Fig 6). In contrast to Mβ and Mγ, the Mα clade of the rubber tree is relatively more active in duplication, and 15 Mα MADS factors are identified. This number is still smaller than that in Arabidopsis (25), but larger than that in rice (13).

**Fig 7:**
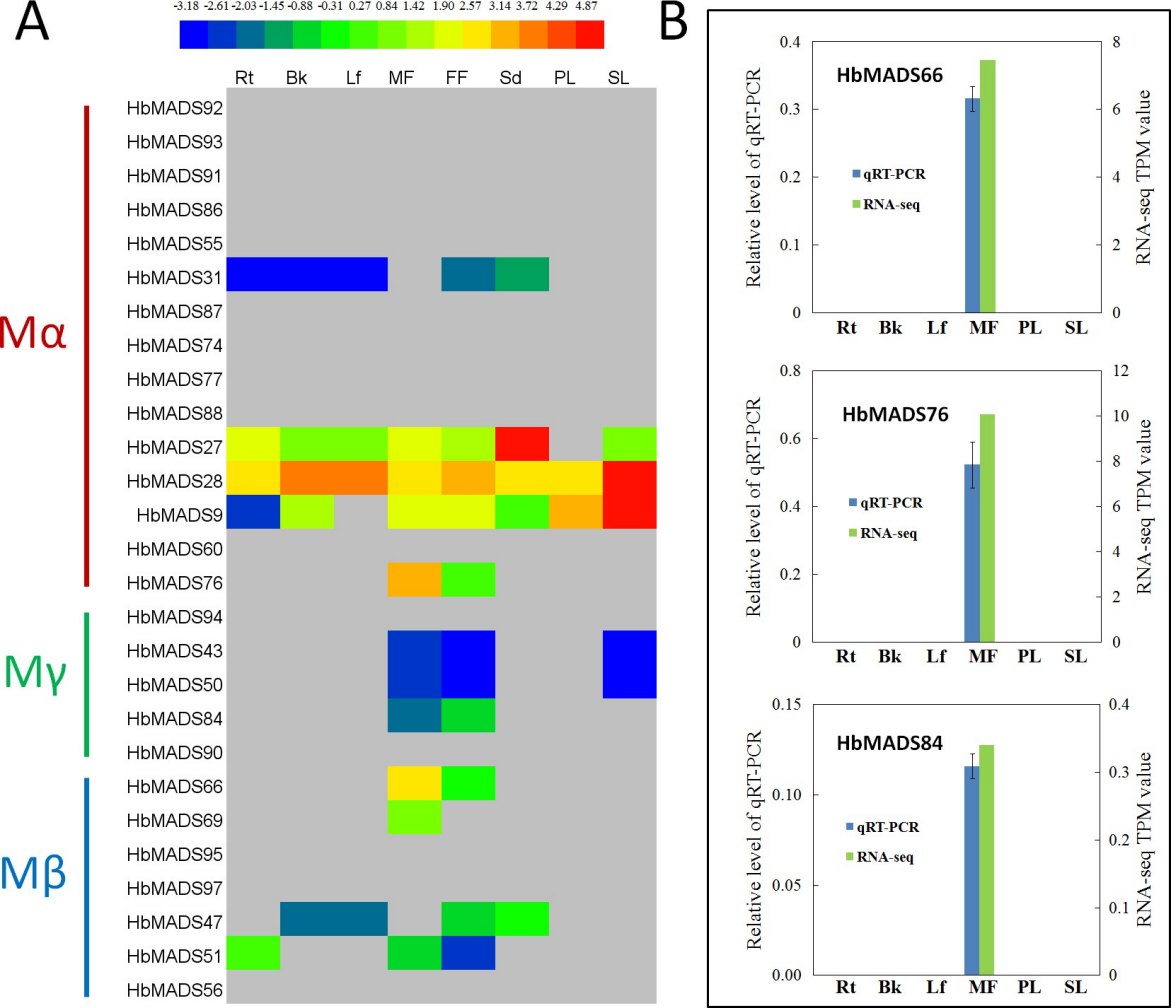
Expression profile of type I MADS-box genes in the rubber tree. A, Expression profiles by transcriptome analysis. B, Real-time quantitative RT-PCR results of representative genes as compared with TPM values in transcriptome analysis. Rt, root; Bk, bark; Lf, leaf; MF, male flower; FF, female flower; PL, primary laticifer; SL, secondary laticifer.

Transcriptome analysis indicates that 55.6% type I genes (15) in the rubber tree are not transcribed in any of the tested tissues (Fig 7A), which is a very high percentage compared to type II MADS genes, in which only 1 out of 70 genes (*HbMADS89*) is not transcribed (Fig 5B). The amino acid sequences of these silent genes are predicted from their genome sequences, and they are probably pseudogenes. These results indicate that type I MADS factor genes in the rubber tree have experienced rapid birth and death in the evolutionary history, in agreement with previous researches [8]. Out of the transcribed type I genes, 80% (12) are expressed in flowers, in which five genes (*HbMADS76*, -*84*, -*66*, -*69*, and -*51*) are specifically expressed in flowers, and *HbMADS76* and -*66* are predominantly expressed in male flowers, which is also confirmed by qRT-PCR analysis (Fig 7B). These results suggest that the most important biological functions of the type I genes are flower development regulation. *HbMADS28* are the most actively expressed gene in the type I subfamily. It is strongly expressed in the secondary laticifer, bark, and leaf, followed by female flower, male flower, seed, root, and primary laticifer. *HbMADS9* is strongly expressed in the secondary laticifers, followed by the primary laticifer, male and female flowers. *HbMADS27* is predominantly expressed in seeds, and expressed in all other tissues, except the primary laticifer.

Taken together, gene duplication events in the type I subfamily of MADS-box transcription factors in the rubber tree are greatly inhibited, especially in Mβ and Mγ clades as compared to the type II genes in the rubber tree. At the same time, 55.6% of the type I genes in the rubber tree are silent, which is only 1.4% (1 out of 70) for the type II genes. These asymmetrical birth and death has resulted in high type II to type I ratio in the rubber tree, which is 2.6. In contrast, the type II to type I ratios in Arabidopsis and rice are only 0.7 and 1.3, respectively (Table 1).

**Table 1:** Number of annotated MADS-box genes present in genomes.

	clades	*A*. *thaliana*	*O*. *sativa*	*H*. *brasilliensis*
Type I	Mα	25	13	16
	Mβ	20	9	7
	Mγ	16	10	5
	subtotal	61	32	27
Type II	MIKC^c^	39	38	58
	MIKC*	6	5	12
	subtotal	43	42	70
Total		104	74	97
Type II/I Ratio		0.7	1.3	2.6

## Discussion

### Duplication pattern of MADS-box gene family in plants

MADS domain of the MADS-box genes are of ancient origin, and can be traced back to the DNA binding subunit A of topoisomerases IIA from an ancestral prokaryote. The primitive MADS-box gene that contained the DNA-binding domain of topoisomerases IIA was duplicated and gave rise to a SRF-like gene and a MEF2-like MADS-boxgene in a MRCA (most recent common ancestor) extant eukaryote approximately 1.5 billion years ago [6]. During the after-birth evolution, MADS-box gene family has experienced distinct birth and death stories in different lineages. In animals and fungi, duplication events are few and only a few MADS-box genes are present, for example, there are only two in *Drosophila melanogaster*, five in human, and four in *Saccharomyces cerevisiae*. These genes play important roles in signal transduction, cell proliferation and differentiation [49].

Plant MADS-box genes diverged much faster than those of animals, and gene duplication and sequence diversification were extensively used for the creation of new genes during plant evolution [50]. Terrestrial plants probably evolved from charophyte green algae [51]. The haplobiontic type characteristic of charophyte green algae were evolved to a gametophyte-dominant life cycle in bryophytes and to a dominant sporophyte-dominant life cycle in vascular plants [52, 53]. During these evolution process, new MADS-box gene members came into being by duplication, and there were at least four genes in the MRCA of land plants, and more than 10 genes in the MRCA of seed plants (spermatophytes) [6].

Modern flowering plants have approximately 50 to 200 MADS-box genes. For example, there are 107 MADS-box genes in Arabidopsis [11], 75 in rice (*Oryza sativa*) [20], 52 in Chinese Jujube [54], 144 in radish [55], 142 in apple [56], 62 in the melon (*Cucumis melo*), 160 in *Brassica rapa* [57], and 180 in bread wheat [58]. In our studies, 97 MADS-box genes were identified.

Plant MADS-box genes can be phylogenetically classified into type I and type II subfamilies. The type I genes can be further divided into three clades (Mα, Mβ, and Mγ), wihle the type II genes are further divided into two clades (MIKC^c^ and MIKC*) [11, 45, 59]. In our research, all the MADS-box genes were classified into type I and type II subfamilies and they are further allocated into conserved subclades.

### MIKC^c^-type MADS-box genes have evolved new function in regulation of laticifer development in the rubber tree

Land plants have a life cycle that switches between a diploid sporophytic and a haploid gametophytic generations. The gametophytic generation of most nonvascular land plants has a considerable morphological diversity, is free-living and can reach a much higher degree of complexity than seed plants. In contrast, the gametophytic generation of seed plants is simple with only a few cells, while their sporophytes have complex morphology, especially the angiosperm flowers, which from sepal to carpel, are all interpreted as modified leaves as reviewed [60]. Classical MIKC-type (MIKC^c^) MADS-box genes were shown to be crucial regulators of sporophytic morphology, especially floral organ identity [3, 61–64]. Therefore, the expansion and diversification of the MIKC^c^-type MADS gene family are considered crucial in diploid sporophytic development and evolution of seed plants [65, 66].

A total of 58 MIKC^c^-type MADS-box genes were identified in the rubber tree, which is a larger number compared to Arabidopsis and rice. These genes were further divided into 15 subclades. Out of which, 56 genes are clustered into 13 subclades of the conserved MIKC^c^-type MADS in monocots and eudicots, which very likely existed already in the MRCA of monocots and eudicots about 200 million years ago [67]. All MADS genes in these subclades are highly duplicated in the rubber tree and are predominantly expressed in flowers, indicating that the conserved roles in regulating sporophytic development have been enhanced in the rubber tree. However, some MIKC^c^ genes are highly expressed in laticifer cells, especially genes in the seven subclades: AGL2-, SQUA-, AGL15-, STMADS11-, AGL12-, GLO-, and DEF-like subclades, in which the number of MIKC^c^-type MADS are twice as many as those in Arabidopsis and rice (Fig 4A and S2 Table). These results indicate that besides the conserved roles of MIKC^c^-type MADS in flower development, the diversified roles also occurred for regulating laticifer development in the rubber tree.

Moreover, one MIKC^c^-type gene member, HbMADS32 is clustered together with a newly identified clade OsMADS32-like MIKC^c^-type MADS factor. This result indicates that OsMADS32-like MIKC^c^ is not rice specific as previous considered [20], and existed before the monocots and eudicots were separated. The absence of the orthologous OsMADS32-like gene in Arabidopsis may have been caused by deletion of this gene in the genome in the evolutionary history after the common ancestor of the rubber tree and Arabidopsis separated into different lineages. RNA-seq and qRT-PCR results indicate that *HbMADS33* is predominantly expressed in female flowers, and also weakly expressed in bark and seed (Fig 4), suggesting its broad role in flower, bark, and seed development.

Interestingly, HbMADS71 outgroups AGL2-like and AGL6-like clades (Fig 4A), suggesting that HbMADS71-like MIKC^c^-type MADS factor may represent the MRCA of the AGL2- and AGL6-like MADS subclade proteins. RNA-seq results show that HbMADS71 is predominantly expressed in male and female flowers (Fig 4B), indicating that this ancient protein is still functioning in floral organ development. Surprisingly, all the AGL2-like and AGL6-like MADS factors are also dominantly expressed in male and female flowers, indicating that the expression pattern and biological function of these two subclade members are highly conserved and can be traced back to the occurrence of the MRCA of the monocots and dicots. Besides AGL2- and AGL6-like clades, most MIKC^c^-type genes are also predominantly expressed in flowers, indicating that MIKC^c^ type genes have biological functions conserved to other plants, e.g. Arabidopsis [43], rice [20], and cotton [68].

The rubber tree is a latex producing crop and contain laticifer networks that facilitate the biosynthesis of polymers of isoprene (cis-1,4-polyisoprene). There are both primary and secondary laticifer cells in the rubber tree [26], and the development of the secondary laticifer system is one of the decisive factors of the rubber yield [69]. Therefore, the MADS-box genes that regulate laticifer development is of great interests to rubber tree biologists and breeders. In our research, most genes in the AGL15-, STMADS11-, and TM3-like MIKC^c^ clades are highly expressed in the primary and/or secondary laticifers, indicating that the biological function of the AGL15-, STMADS11-, and TM3-like MIKC^c^ clades are distinct to their orthologous genes in Arabidopsis and rice. In total, 11 genes including the previously reported *HbMADS2*, *-3*, and *-4* [27] are highly expressed in laticifers. These results indicates that MIKC^c^-type MADS have been recruited in regulating laticifer development and rubber biosynthesis in the rubber tree.

### Typical and atypical MIKC*-type MADS-box genes in angiosperms

In contrast to the well known roles of MIKC^c^-type MADS genes in regulating sporophyte development, the MIKC*-type MADS genes are well known for their regulation of haploid gametophyte development in land plants including the earliest diverging land plants liverworts [70], bryophytes and lycophytes [71], and the more popular plants Arabidopsis and rice [45, 46, 72] and other land plants [70]. Phylogenetic analysis revealed that MIKC*-type MADS are extraordinary conserved compared to MIKC^c^ genes, and a conserved network operates in the gametophytes of all tested model organisms [45]. Moreover, MIKC* genes probably evolved from an ancestral MIKC^c^-type gene by a duplication in the Keratin-like region [45].

We identified a total of 12 MIKC* genes in the rubber tree, which is a much larger number than that in Arabidopsis (5) and rice (5) and is a result of active duplication during evolution, similar to the MIKC^c^-type MADS-box factors. Phylogenetic analysis indicates that there are both conserved and diverged MIKC* genes in the rubber tree compared to Arabidopsis and rice. The conserved HbMADS are clustered in the typical S-clade and P-clade MIKC* (Fig 5A). Genes in the typical P- and S- clades are considered as genuine MIKC*-type genes and are specifically expressed in gametophyte development [44, 45] and serve as conserved roles during land plant evolution [45]. This role has been conserved since the divergence of monocots and eudicots, roughly 150 million years ago [46].

However, we also identified nonconserved MIKC*-type MADS genes in the rubber tree. They are clustered in atypical clades in the S- and P-clades (Fig 5A), respectively. In previous studies in rice, although five genes were grouped with MIKC*-type genes [20], two (OsMADS37 and -65) were regarded as not genuine MIKC* and omitted from analysis [46]. We included these two proteins in our analysis and revealed that OsMADS37 and -65 together with four HbMADS (HbMADS1, -82, -83, -85, and -89) are clustered as an atypical S-clade with high bootstrap supports (Fig 5A). Similarly, P-clade MIKC* also includes an atypical subgroup HbMADS20, which outgroups the typical P-clade members from the monocots and eudicots, which suggests that HbMADS20 may represent the MRCA of the P-clade MIKC*-type MADS-box proteins of monocots and eudicots.

The typical S-clade and P-clade MIKC* members in rice and Arabidopsis have conserved expression pattern and are mainly involved in pollen development and pollen tube growth [10, 46–48]. It is interesting that the two typical S-clade members in the rubber tree (*HbMADS62* and *-81*) are also specifically expressed in male flowers (Fig 5B), suggesting their conserved function in pollen development. Three atypical S-clade members, *HbMADS82*, *-83*, and *-85* are also specifically expressed in flowers, especially in male flowers.

*HbMADS1* is the first characterized MADS-box gene in the rubber tree [27]. This gene is clustered in the atypical S-clade MIKC* (Fig 5A) and is predominantly expressed in the secondary laticifer and the primary laticifer (Fig 5B). It is also expressed in leaf, bark, flowers, and seeds at low levels, probably due to existence of laticifers in all the tested tissues, therefore, our result does not rule out that *HbMADS1* is specifically expressed in the laticifer cells. These results suggest that the atypical S-clade MIKC* genes, such as *HbMADS1*, may have evolved biological function divergent to their orthologous genes in Arabidopsis and rice, it may represent the most functionally diverged MIKC*-type MADS-box gene.

P-clade MIKC* genes in the rubber tree also evolved biological roles diverged to their orthologous genes in Arabidopsis and rice. The expression patterns of the four typical P-clade members are more or less similar except *HbMADS15*, most of them (*HbMADS17*, *-18*, and *-21*) seem to be constitutively expressed in all tissues, but *HbMADS15* is highly expressed in leaf and female flowers and relatively weak expressed in other tissues (Fig 5B). There is also an atypical P-clade member *HbMADS20*, which is specifically expressed in the secondary laticifer cells. These results suggest that the P-clade MIKC* genes in the rubber tree may play more complex roles than their orthologous genes in Arabidopsis and rice.

Taken together, there are both typical and atypical MIKC*-type MADS genes in both S-clade and P-clade in the rubber tree. The typical S-clade MIKC* genes are more conserved than the typical P-clade MIKC* genes, and are specifically expressed in male flowers and play conserved biological function as their orthologous genes in rice and Arabidopsis. The typical P-clade MIKC* genes are expressed in all tissues and have evolved roles in regulating the development of all tissues, especially *HbMADS15*, which are predominantly expressed in leaf and female flowers, most divergent to its orthologous genes in rice and Arabidopsis that play conserved role in gametophyte development. Moreover, the atypical MIKC* factors (e.g. *HbMADS1* in S-clade, and *HbMADS20* in P-clade) are more diverged and facilitate functions required in laticifer development and rubber biosynthesis, which is not necessary in Arabidopsis and rice.

### Type I genes are mostly silent and have experienced high death rate

While type II MADS-box genes were duplicated during the whole genome duplication that occurred early in angiosperm evolution, many type I genes show signs of intrachromosomal duplication events happening gradually and more recent in evolution (Martinez-Castilla and Alvarez-Buylla, 2003), which may have resulted in great difference in type I gene numbers among angiosperm species. In most angiosperm plants, type I MADS-box genes constitutes approximately 40% of the MADS-box gene family. However, rubber tree has a much smaller percentage (27%) of type I genes and most of them are silent, suggesting that type I genes in the rubber tree have been experiencing low birth rate and high death rate in the evolutionary history. There is similar phenomenon in some other species, for example, in a transcriptome database analysis, only one out of the 28 MADS-box genes in the orchid *Erycina pusilla* was classified into type I [73]. By enumerating all available functional and non-functional MADS-box genes in Arabidopsis and rice, Nam et al. found that type I genes have experienced faster birth and death evolution than type II genes in angiosperms [8]. The type I genes in the rubber tree may have been experiencing even rapid death rate than Arabiodpsis and rice.

## Supporting information

S1 Fig Phylogenetic analysis of type II MADS-box proteins in the rubber tree. (TIF)

S2 Fig Deletion mutations occurred in *HbMADS85*. (TIF)

S1 Table Primers used in real-time qRT-PCR analysis. (XLSX)

S2 Table Statistic analysis of assembled transcriptomes of different tissues. (DOCX)

S3 Table MADS-box genes identified in the rubber tree genome and transcriptomes. (XLSX)

S4 Table MADS-box genes in Arabidopsis used in this research. (DOCX)

S5 Table MADS-box genes in rice used in this research. (DOCX)

S6 Table MADS-box genes in other species used in this research. (DOCX)
