# Supplementary figures and images for: Asymmetric birth and death of type I and type II MADS-box gene subfamilies in the rubber tree facilitating laticifer development
Source: PLoS One. 2019 Apr 1;14(4):e0214335. doi: 10.1371/journal.pone.0214335 (PMC6443149; doi:10.1371/journal.pone.0214335)

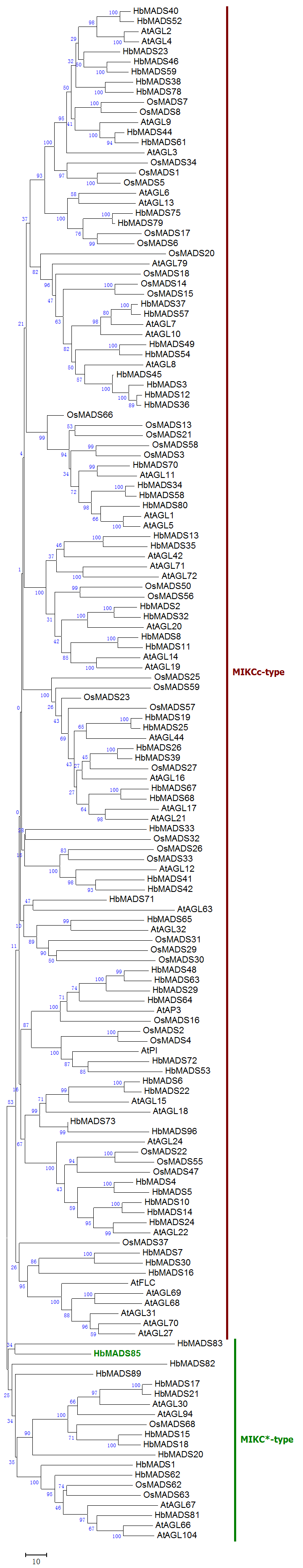

Supplement: S1 Fig — (TIF) [file pone.0214335.s001.tif]

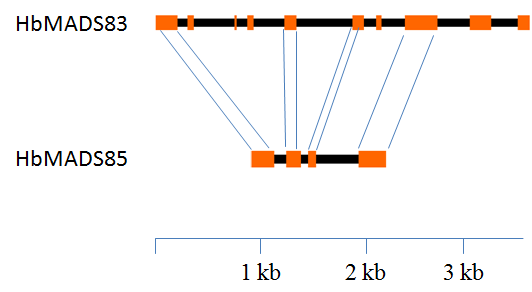

Supplement: S2 Fig — (TIF) [file pone.0214335.s002.tif]
