# Supplementary material for: Asymmetric birth and death of type I and type II MADS-box gene subfamilies in the rubber tree facilitating laticifer development
Source: PLoS One. 2019 Apr 1;14(4):e0214335. doi: 10.1371/journal.pone.0214335 (PMC6443149; doi:10.1371/journal.pone.0214335)
Supplement: S2 Table — (DOCX) [file pone.0214335.s004.docx]

**S2_Table**. Statistics of the transcriptomes of the rubber tree tissues

|  | Trinity transcripts | | | Trinity 'genes' | | | GC% |
| --- | --- | --- | --- | --- | --- | --- | --- |
|  | Number | N50 | Total bases | number | N50 | Total bases |  |
| root | 120,812 | 1492 | 113,579,521 | 64,939 | 1223 | 45,433,653 | 41.45 |
| bark | 120,812 | 1492 | 113,579,521 | 64,939 | 1223 | 45,433,653 | 41.45 |
| leaf | 114,367 | 1842 | 131,610,366 | 54,804 | 1527 | 43,237,440 | 40.56 |
| Male flower | 129,810 | 1803 | 144,039,610 | 68,837 | 1415 | 53,457,536 | 40.32 |
| Female flower | 144,332 | 1877 | 173,116,269 | 65,689 | 1621 | 55,840,318 | 40.11 |
| Seed | 173,664 | 1907 | 211,445,231 | 64,408 | 1367 | 48,415,395 | 39.84 |
| Primary laticifer | 61,766 | 1209 | 52,603,884 | 36,944 | 1055 | 27,076,287 | 42.05 |
| Secondary laticifer | 50,504 | 1815 | 60,516,285 | 28,726 | 1602 | 27,101,997 | 42.03 |
| Whole plant | 388,766 | 1801 | 421,455,188 | 158,136 | 1317 | 121,851,161 | 39.70 |
