# Supplementary material for: Asymmetric birth and death of type I and type II MADS-box gene subfamilies in the rubber tree facilitating laticifer development
Source: PLoS One. 2019 Apr 1;14(4):e0214335. doi: 10.1371/journal.pone.0214335 (PMC6443149; doi:10.1371/journal.pone.0214335)
Supplement: S4 Table — (DOCX) [file pone.0214335.s006.docx]

**S4_Table.** List of 105 MADS-box genes identified in *Arabidopsis thaliana* ecotype Columbia

| **No.** | **Gene name** | **Accession Number** | |  | **Protein** | | | **Types** | **Group** | **Synonym** |
| --- | --- | --- | --- | --- | --- | --- | --- | --- | --- | --- |
|  |  | **GeneBank** | **Location** |  | **Size (aa)** | **MW (KD)** | **PI** |  |  |  |
| 1 | AGL1 | NP_191437.1 | At3g58780 |  | 248 | 28335.8 | 8.93 | II | MIKC^c^ | SHP1 |
| 2 | AGL2 | NP_568322.1 | At5g15800 |  | 251 | 28656.5 | 8.13 | II | MIKC^c^ | SEP1 |
| 3 | AGL3 | AAD20073.1 | At2g03710 |  | 258 | 29573.3 | 8.78 | II | MIKC^c^ |  |
| 4 | AGL4 | NP_186880.1 | At3g02310 |  | 250 | 28577.5 | 8.02 | II | MIKC^c^ | SEP2 |
| 5 | AGL5 | NP_565986.1 | At2g42830 |  | 246 | 28128.7 | 9.19 | II | MIKC^c^ | SHP2 |
| 6 | AGL6 | NP_182089.1 | At2g45650 |  | 252 | 28744.5 | 7.35 | II | MIKC^c^ |  |
| 7 | AGL7 | NP_177074.1 | At1g69120 |  | 256 | 30182.5 | 8.07 | II | MIKC^c^ | AP1 |
| 8 | AGL8 | NP_568929.1 | At5g60910 |  | 242 | 27536.4 | 9.45 | II | MIKC^c^ | FUL |
| 9 | AGL9 | NP_850953.1 | At1g24260 |  | 250 | 28966.9 | 7.78 | II | MIKC^c^ | SEP3 |
| 10 | AGL10 | Q39081.3 | At1g26310 |  | 255 | 30187.4 | 7.80 | II | MIKC^c^ | CAL |
| 11 | AGL11 | NP_001329611.1 | At4g09960 |  | 240 | 27391.0 | 9.29 | II | MIKC^c^ |  |
| 12 | AGL12 | NP_565022.1 | At1g71692 |  | 211 | 23918.7 | 6.85 | II | MIKC^c^ |  |
| 13 | AGL13 | NP_191671.1 | At3g61120 |  | 244 | 27967.6 | 6.47 | II | MIKC^c^ |  |
| 14 | AGL14 | Q38838.2 | At4g11880 |  | 221 | 25492.1 | 9.09 | II | MIKC^c^ |  |
| 15 | AGL15 | NP_196883.1 | At5g13790 |  | 268 | 30329.0 | 8.11 | II | MIKC^c^ |  |
| 16 | AGL16 | A2RVQ5.1 | At3g57230 |  | 240 | 27428.1 | 6.97 | II | MIKC^c^ |  |
| 17 | AGL17 | NP_179848.1 | At2g22630 |  | 227 | 26313.9 | 8.83 | II | MIKC^c^ |  |
| 18 | AGL18 | NP_191298.1 | At3g57390 |  | 256 | 28874.5 | 5.78 | II | MIKC^c^ |  |
| 19 | AGL19 | NP_194026.1 | At4g22950 |  | 219 | 25048.5 | 9.65 | II | MIKC^c^ |  |
| 20 | AGL20 | NP_182090.1 | At2g45660 |  | 214 | 24533.0 | 8.95 | II | MIKC^c^ | SOC1 |
| 21 | AGL21 | NP_195507.1 | At4g37940 |  | 228 | 26411.0 | 8.88 | II | MIKC^c^ |  |
| 22 | AGL22 | NP_179840.2 | At2g22540 |  | 240 | 26896.2 | 5.30 | II | MIKC^c^ | SVP |
| 23 | AGL23 | NP_176715.1 | At1g65360 |  | 226 | 25572.2 | 9.66 | I | Mα |  |
| 24 | AGL24 | NP_194185.1 | At4g24540 |  | 220 | 25063.7 | 8.00 | II | MIKC^c^ |  |
| 25 | AGL27 | NP_177833.3 | At1g77080 |  | 196 | 22119.2 | 5.14 | II | MIKC^c^ | FLM |
| 26 | AGL28 | NP_171660.1 | At1g01530 |  | 247 | 28029.5 | 9.31 | I | Mα |  |
| 27 | AGL29 | NP_180991.1 | At2g34440 |  | 172 | 19753.3 | 6.98 | I | Mα |  |
| 28 | AGL30 | NP_001318186.1 | At2g03060 |  | 381 | 43248.5 | 5.97 | II | MIKC* |  |
| 29 | AGL31 | NP_001078798.1 | At5g65050 |  | 178 | 19980.7 | 6.25 | II | MIKC^c^ |  |
| 30 | AGL32 | NP_001330404.1 | At5g23260 |  | 268 | 31635.7 | 6.38 | II | MIKC^c^ | TT16 |
| 31 | AGL34 | NP_850888.2 | At5g26580 |  | 345 | 39581.9 | 5.74 | I | Mᵞ |  |
| 32 | AGL35 | NP_850882.2 | At5g26630 |  | 218 | 25123.2 | 10.09 | I | Mᵞ |  |
| 33 | AGL36 | NP_850880.2 | At5g26650 |  | 366 | 38211.5 | 6.37 | I | Mᵞ |  |
| 34 | AGL37 | NP_176712.1 | At1g65330 |  | 279 | 31953.4 | 5.12 | I | Mᵞ |  |
| 35 | AGL38 | NP_176709.2 | At1g65300 |  | 278 | 31658.0 | 5.22 | I | Mᵞ |  |
| 36 | AGL39 | NP_198065.2 | At5g27130 |  | 306 | 33384.7 | 4.25 | I | Mα |  |
| 37 | AGL40 | NP_195377.1 | At4g36590 |  | 248 | 28470.2 | 9.13 | I | Mα |  |
| 38 | AGL42 | Q9FIS1.1 | At5g62165 |  | 210 | 24673.4 | 9.65 | II | MIKC^c^ |  |
| 39 | AGL43 | NP_198838.2 | At5g40220 |  | 324 | 37076.7 | 7.26 | I | Mᵝ |  |
| **No.** | **Gene name** | **Accession Number** | |  | **Protein** | | | **Types** | **Group** | **Synonym** |
|  |  | **GeneBank** | **Location** |  | **Size (aa)** | **MW (KD)** | **PI** |  |  |  |
| 40 | AGL44 | NP_179033.1 | At2g14210 |  | 234 | 26802.8 | 10.00 | II | MIKC^c^ | ANR1 |
| 41 | AGL46 | NP_180438.2 | At2g28700 |  | 329 | 38020.1 | 5.45 | I | Mᵞ |  |
| 42 | AGL47 | NP_200380.1 | At5g55690 |  | 277 | 31700.0 | 7.30 | I | Mᵝ |  |
| 43 | AGL48 | NP_181550.1 | At2g40210 |  | 371 | 42414.2 | 8.83 | I | Mᵞ |  |
| 44 | AGL49 | NP_176212.1 | At1g60040 |  | 284 | 32148.5 | 5.89 | I | Mᵝ |  |
| 45 | AGL50 | NP_176190.1 | At1g59810 |  | 283 | 32157.1 | 6.14 | I | Mᵝ |  |
| 46 | AGL51 | NP_974500.1 | At4g02235 |  | 196 | 22478.4 | 9.91 | I | Mᵝ |  |
| 47 | AGL52 | NP_192864.1 | At4g11250 |  | 329 | 37852.4 | 7.85 | I | Mᵝ |  |
| 48 | AGL53 | NP_198059.1 | At5g27070 |  | 287 | 32684.0 | 8.10 | I | Mᵝ |  |
| 49 | AGL54 | NP_198061.2 | At5g27090 |  | 187 | 21737.8 | 8.74 | I | Mᵝ |  |
| 50 | AGL55 | NP_176288.1 | At1g60920 |  | 191 | 21422.0 | 6.21 | I | Mα |  |
| 51 | AGL56 | NP_176285.1 | At1g60880 |  | 201 | 22659.3 | 5.28 | I | Mα |  |
| 52 | AGL57 | NP_187060.1 | At3g04100 |  | 207 | 24099.1 | 10.08 | I | Mα |  |
| 53 | AGL58 | NP_174167.1 | At1g28450 |  | 185 | 21145.4 | 9.92 | I | Mα |  |
| 54 | AGL59 | NP_174168.1 | At1g28460 |  | 182 | 20678.8 | 9.76 | I | Mα |  |
| 55 | AGL60 | NP_177379.1 | At1g72350 |  | 224 | 25513.1 | 8.88 | I | Mα |  |
| 56 | AGL61 | NP_850058.1 | At2g24840 |  | 264 | 23849.6 | 9.13 | I | Mα |  |
| 57 | AGL62 | NP_200852.1 | At5g60440 |  | 299 | 34587.8 | 8.72 | I | Mα |  |
| 58 | AGL63 | NP_174399.2 | At1g31140 |  | 213 | 24931.6 | 9.69 | II | MIKC^c^ |  |
| 59 | AGL64 | NP_001077625.1 | At1g29962 |  | 185 | 21206.3 | 8.88 | I | Mα |  |
| 60 | AGL65 | Q7X9I0.1 | AtT1g18750 |  | 389 | 44877.5 | 6.08 | I | Mα |  |
| 61 | AGL66 | NP_177921.2 | At1g77980 |  | 332 | 38280.6 | 4.66 | II | MIKC* |  |
| 62 | AGL67 | NP_001117616.1 | At1g77950 |  | 252 | 29245.2 | 5.48 | II | MIKC* |  |
| 63 | AGL68 | Q683D7.2 | At5g65080 |  | 198 | 22342.9 | 9.56 | II | MIKC^c^ |  |
| 64 | AGL69 | AED97997.1 | At5g65070 |  | 232 | 26492.6 | 6.83 | II | MIKC^c^ |  |
| 65 | AGL70 | AED97993.1 | At5g65060 |  | 196 | 22129.5 | 7.98 | II | MIKC^c^ |  |
| 66 | AGL71 | Q9LT93 | At5g51870 |  | 207 | 24078.7 | 9.68 | II | MIKC^c^ |  |
| 67 | AGL72 | Q9FLH5.1 | At5g51860 |  | 211 | 24633.8 | 9.87 | II | MIKC^c^ |  |
| 68 | AGL73 | NP_198678.1 | At5g38620 |  | 349 | 38849.0 | 4.20 | I | Mα |  |
| 69 | AGL74 | NP_175249.1 | At1g48150 |  | 184 | 20946.5 | 7.71 | I | Mα |  |
| 70 | AGL75 | NP_198936.1 | At5g41200 |  | 330 | 33462.8 | 8.18 | I | Mᵝ |  |
| 71 | AGL76 | NP_198828.1 | At5g40120 |  | 385 | 44018.0 | 9.11 | I | Mᵝ |  |
| 72 | AGL77 | NP_198690.1 | At5g38740 |  | 426 | 48460.7 | 7.55 | I | Mᵝ |  |
| 73 | AGL78 | NP_201336.1 | At5g65330 |  | 341 | 38921.5 | 7.71 | I | Mᵝ |  |
| 74 | AGL79 | NP_189645.2 | At3g30260 |  | 249 | 28061.1 | 9.84 | II | MIKC^c^ |  |
| 75 | AGL80 | AED95709.1 | At5g48670 |  | 321 | 36824.5 | 6.49 | I | Mᵞ |  |
| 76 | AGL81 | NP_198791.1 | At5g39750 |  | 356 | 40177.9 | 5.66 | I | Mᵝ |  |
| 77 | AGL82 | NP_200697.1 | At5g58890 |  | 294 | 34165.9 | 8.79 | I | Mᵝ |  |
| 78 | AGL83 | NP_199760.1 | At5g49490 |  | 285 | 32425.0 | 8.50 | I | Mα |  |
| 79 | AGL84 | NP_199753.3 | At5g49420 |  | 334 | 37396.5 | 4.23 | I | Mα |  |
| 80 | AGL85 | NP_175874.1 | At1g54760 |  | 161 | 18175.5 | 4.93 | I | Mα |  |
| **No.** | **Gene name** | **Accession Number** | |  | **Protein** | | | **Types** | **Group** | **Synonym** |
|  |  | **GeneBank** | **Location** |  | **Size (aa)** | **MW (KD)** | **PI** |  |  |  |
| 81 | AGL86 | NP_174444.1 | At1g31630 |  | 339 | 38280.2 | 6.24 | I | Mᵞ |  |
| 82 | AGL88 | AAO74631.1 | At2g11990 |  | 129 | 15330.8 | 6.42 | I | Mα |  |
| 83 | AGL89 | NP_198110.1 | At5g27580 |  | 223 | 25589.9 | 8.38 | I | Mᵝ |  |
| 84 | AGL90 | NP_198148.2 | At5g27960 |  | 320 | 38294.5 | 7.77 | I | Mᵞ |  |
| 85 | AGL91 | NP_187320.1 | At3g66656 |  | 178 | 20374.0 | 6.91 | I | Mα |  |
| 86 | AGL92 | NP_174445.1 | At1g31640 |  | 464 | 56480.8 | 5.21 | I | Mᵞ |  |
| 87 | AGL93 | NP_198047.1 | At5g26950 |  | 289 | 32755.1 | 7.96 | I | Mᵝ |  |
| 88 | AGL94 | NP_177113.3 | At1g69540 |  | 344 | 39340.8 | 6.35 | II | MIKC* |  |
| 89 | AGL95 | NP_179168.2 | At2g15660 |  | 250 | 44386.6 | 7.19 | I | Mᵞ |  |
| 90 | AGL96 | NP_196268.1 | At5g06500 |  | 242 | 27389.0 | 4.95 | I | Mᵞ |  |
| 91 | AGL97 | NP_175144.1 | At1g46408 |  | 266 | 29878.8 | 4.01 | I | Mα |  |
| 92 | AGL98 | NP_198797.1 | At5g39810 |  | 329 | 37288.6 | 5.64 | I | Mᵝ |  |
| 93 | AGL99 | NP_196084.1 | At5g04640 |  | 322 | 36553.6 | 4.14 | I | Mα |  |
| 94 | AGL100 | NP_173175.1 | At1g17310 |  | 217 | 24919.7 | 9.13 | I | Mα |  |
| 95 | AGL101 | NP_198057.1 | At5g27050 |  | 120 | 14188.5 | 9.67 | I | Mᵝ |  |
| 96 | AGL102 | NP_175207.1 | At1g47760 |  | 184 | 21322.7 | 9.85 | I | Mα |  |
| 97 | AGL103 | NP_188495.1 | At3G18650 |  | 386 | 43175.2 | 8.98 | I | Mᵝ |  |
| 98 | AGL104 | NP_173632.1 | At1g22130 |  | 335 | 38219.7 | 4.81 | II | MIKC* |  |
| 99 | AGL105 | NP_001119325.1 | At5g37415 |  | 237 | 28126.5 | 9.97 | I | Mᵝ |  |
| 100 | AG | NP_001190766.1 | At4g18960 |  | 238 | 27161.8 | 9.32 | II | MIKC^c^ |  |
| 101 | AP3 | NP_191002.1 | At3g54340 |  | 232 | 27340.9 | 8.48 | II | MIKC^c^ |  |
| 102 | FLC | NP_196576.1 | At5g10140 |  | 196 | 21865.2 | 7.95 | II | MIKC^c^ |  |
| 103 | PI | NP_197524.1 | At5g20240 |  | 208 | 24046.9 | 8.98 | II | MIKC^c^ |  |

**Note:**

AGL33 (Rita *et al*, 2007)and AGL45 (Lucie *et al*, 2003) originally identified in *Arabidopsis thaliana* ecotype Landsperg and *Arabidopsis halleri subsp. tATrica* respectively, are not found in the ecotype Colombia, and thus not included in this list.

AGL26, AGL41 and AGL87 originally identified in *Arabidopsis thaliana* ecotype Colombia (Rita *et al*, 2007) are not MADS box gene by CD-search (https://www.ncbi.nlm.nih.gov/Structure/bwrpsb/bwrpsb.cgi).
