# Supplementary material for: Asymmetric birth and death of type I and type II MADS-box gene subfamilies in the rubber tree facilitating laticifer development
Source: PLoS One. 2019 Apr 1;14(4):e0214335. doi: 10.1371/journal.pone.0214335 (PMC6443149; doi:10.1371/journal.pone.0214335)
Supplement: S5 Table — (DOCX) [file pone.0214335.s007.docx]

**S5_Table.** List of 74 MADS-box genes identified in rice and used in this research.

| **No.** | **Gene name** | **Accession Number** | |  | **Protein** | | | **Types** | **Group** |
| --- | --- | --- | --- | --- | --- | --- | --- | --- | --- |
|  |  | **GeneBank** | **TIGR/RAGP** |  | **Size (aa)** | **MW (KD)** | **PI** |  |  |
| 1 | OsMADS1 | XP_015628585.1 | LOC_Os03g11614 |  | 257 | 29528.2 | 6.85 | II | MIKC^c^ |
| 2 | OsMADS2 | Q40702.1 | LOC_Os01g66030 |  | 209 | 24157.6 | 7.21 | II | MIKC^c^ |
| 3 | OsMADS3 | Q40704.1 | LOC_Os01g10504 |  | 287 | 27140.6 | 9.31 | II | MIKC^c^ |
| 4 | OsMADS4 | Q40703.3 | LOC_Os05g34940 |  | 210 | 24908.5 | 8.64 | II | MIKC^c^ |
| 5 | OsMADS5 | Q0DEB8.1 | LOC_Os06g06750 |  | 225 | 25993.6 | 6.96 | II | MIKC^c^ |
| 6 | OsMADS6 | XP_015623947.1 | LOC_Os02g45770 |  | 250 | 28437.4 | 8.86 | II | MIKC^c^ |
| 7 | OsMADS7/45 | Q0J466.2 | LOC_Os08g41950 |  | 310 | 28769.8 | 8.70 | II | MIKC^c^ |
| 8 | OsMADS8/24 | XP_015610824.1 | LOC_Os09g32948 |  | 248 | 28527.5 | 8.73 | II | MIKC^c^ |
| 9 | OsMADS13 | XP_015618486.1 | LOC_Os12g10540 |  | 270 | 30268.5 | 9.03 | II | MIKC^c^ |
| 10 | OsMADS14 | Q10CQ1.2 | LOC_Os03g54160 |  | 253 | 28422.5 | 8.98 | II | MIKC^c^ |
| 11 | OsMADS15 | Q6Q9I2.2 | LOC_Os07g01820 |  | 267 | 30390.7 | 9.00 | II | MIKC^c^ |
| 12 | OsMADS16 | Q944S9.2 | LOC_Os06g49840 |  | 224 | 25463.8 | 8.14 | II | MIKC^c^ |
| 13 | OsMADS17 | ACX35552.1 | LOC_Os04g49150 |  | 254 | 28802.6 | 8.45 | II | MIKC^c^ |
| 14 | OsMADS18/28 | Q0D4T4.1 | LOC_Os07g41370 |  | 249 | 28284.8 | 8.80 | II | MIKC^c^ |
| 15 | OsMADS20 | Q2QQA3.2 | LOC_Os12g31748 |  | 233 | 26119.7 | 7.02 | II | MIKC^c^ |
| 16 | OsMADS21 | XP_015621182.1 | LOC_Os01g66290 |  | 265 | 29393.1 | 8.13 | II | MIKC^c^ |
| 17 | OsMADS22 | Q9XJ66.1 | LOC_Os02g52340 |  | 228 | 25592.6 | 5.39 | II | MIKC^c^ |
| 18 | OsMADS23 | Q6VAM4.1 | LOC_Os08g33488 |  | 159 | 18361.9 | 9.40 | II | MIKC^c^ |
| 19 | OsMADS25 | Q84NC5.2 | LOC_Os04g23910 |  | 227 | 26141.7 | 8.48 | II | MIKC^c^ |
| 20 | OsMADS26 | Q0J8G8.1 | LOC_Os08g02070 |  | 222 | 25217.2 | 6.94 | II | MIKC^c^ |
| 21 | OsMADS27 | Q6EP49.2 | LOC_Os02g36924 |  | 240 | 27422.1 | 8.98 | II | MIKC^c^ |
| 22 | OsMADS29 | Q6H711.1 | LOC_Os02g07430 |  | 260 | 28993.9 | 6.40 | II | MIKC^c^ |
| 23 | OsMADS30 | Q655V4.1 | LOC_Os06g45650 |  | 221 | 25780.9 | 7.02 | II | MIKC^c^ |
| 24 | OsMADS31 | XP_015635703.1 | LOC_Os04g52410 |  | 241 | 28098.4 | 7.65 | II | MIKC^c^ |
| 25 | OsMADS32 | XP_015642650.1 | LOC_Os01g52680 |  | 196 | 22531.8 | 7.70 | II | MIKC^c^ |
| 26 | OsMADS33 | Q2QW55.2 | LOC_Os12g10520 |  | 202 | 23232.9 | 9.35 | II | MIKC^c^ |
| 27 | OsMADS34 | Q6Q9H6.2 | LOC_Os03g54170 |  | 239 | 26880.5 | 7.04 | II | MIKC^c^ |
| 28 | OsMADS37 | XP_015649438.1 | LOC_Os08g41960 |  | 183 | 20764.8 | 9.84 | II | MIKC^c^ |
| 29 | OsMADS47 | Q5K4R0.2 | LOC_Os03g08754 |  | 246 | 27456.9 | 6.06 | II | MIKC^c^ |
| 30 | OsMADS50 | Q9XJ60.1 | LOC_Os03g03100 |  | 116 | 26180.9 | 8.89 | II | MIKC^c^ |
| 31 | OsMADS55 | Q69TG5.2 | LOC_Os06g11330 |  | 223 | 27543.2 | 5.02 | II | MIKC^c^ |
| 32 | OsMADS56 | P0C5B2.1 | LOC_Os10g39130 |  | 233 | 26305.1 | 9.71 | II | MIKC^c^ |
| 33 | OsMADS57 | Q6Z6W2.2 | LOC_Os02g49840 |  | 241 | 27236.0 | 8.70 | II | MIKC^c^ |
| 34 | OsMADS58 | XP_015638060.1 | LOC_Os05g11414 |  | 343 | 38961.0 | 7.17 | II | MIKC^c^ |
| 35 | OsMADS59 | XP_015641670.1 | LOC_Os06g23950 |  | 228 | 26266.6 | 8.52 | II | MIKC^c^ |
| 36 | OsMADS61 | CAE75993.1 | LOC_Os04g38770 |  | 99 | 11297.0 | 9.66 | II | MIKC^c^ |
| 37 | OsMADS62 | CAX11684.1 | LOC_Os08g38590 |  | 339 | 37244.9 | 4.65 | II | MIKC* |
| 38 | OsMADS63 | XP_015641968.2 | LOC_Os06g11970 |  | 431 | 47910.8 | 4.65 | II | MIKC* |
| 39 | OsMADS64 | XP_015637115.1 | LOC_Os04g31804 |  | 249 | 27939.6 | 6.76 | I | Mα |
| **No.** | **Gene name** | **Accession Number** | |  | **Protein** | | | **Types** | **Group** |
|  |  | **GeneBank** | **TIGR/RAGP** |  | **Size (aa)** | **MW (KD)** | **PI** |  |  |
| 40 | OsMADS65 | Q9XJ61.1 | LOC_Os01g69850 |  | 164 | 18363.5 | 9.97 | II | MIKC* |
| 41 | OsMADS66 | XP_025881105.1 | LOC_Os05g11380 |  | 162 | 18058.3 | 7.53 | II | MIKC^c^ |
| 42 | OsMADS67 | ABA98556.1 | LOC_Os12g31010 |  | 53 | 6364.6 | 10.91 | II | MIKC^c^ |
| 43 | OsMADS68 | CAX11685.1 | LOC_Os11g43740 |  | 385 | 42267.5 | 6.27 | II | MIKC* |
| 44 | OsMADS69 | AAQ56571.1 | LOC_Os08g20440 |  | 184 | 20099.0 | 8.93 | I | Mα |
| 45 | OsMADS70 | XP_015638580.1 | LOC_Os05g23780 |  | 218 | 24082.5 | 8.32 | I | Mα |
| 46 | OsMADS71 | XP_015642726.1 | LOC_Os06g22760 |  | 238 | 27025.1 | 9.80 | I | Mα |
| 47 | OsMADS72 | ABF95010.1 | LOC_Os03g14850 |  | 185 | 20579.8 | 9.74 | I | Mα |
| 48 | OsMADS73 | XP_015619582.1 | LOC_Os12g21850 |  | 194 | 21194.4 | 10.96 | I | Mα |
| 49 | OsMADS74 | ABA97560.1 | LOC_Os12g21880 |  | 119 | 12828.7 | 8.76 | I | Mα |
| 50 | OsMADS75 | BAD45640.1 | LOC_Os06g30810 |  | 210 | 22761.4 | 4.89 | I | Mα |
| 51 | OsMADS76 | XP_015642715.1 | LOC_Os06g30830 |  | 190 | 20346.1 | 10.71 | I | Mα |
| 52 | OsMADS77 | XP_015611505.1 | LOC_Os09g02780 |  | 185 | 20132.1 | 4.94 | I | Mα |
| 53 | OsMADS78 | XP_015611069.1 | LOC_Os09g02830 |  | 210 | 23180.7 | 10.42 | I | Mα |
| 54 | OsMADS79 | XP_015640475.1 | LOC_Os01g74440 |  | 208 | 22916.3 | 10.40 | I | Mα |
| 55 | OsMADS80 | XP_025878655.1 | LOC_Os02g06860 |  | 307 | 33161.5 | 6.90 | I | Mα |
| 56 | OsMADS81 | XP_015634782.1 | LOC_Os04g24790 |  | 209 | 23213.0 | 9.75 | I | Mᵞ |
| 57 | OsMADS82 | XP_015634783.1 | LOC_Os04g24800 |  | 209 | 23256.0 | 9.70 | I | Mᵞ |
| 58 | OsMADS83 | XP_015634784.1 | LOC_Os04g24810 |  | 209 | 23576.7 | 10.29 | I | Mᵞ |
| 59 | OsMADS84 | XP_015634792.1 | LOC_Os04g25870 |  | 209 | 23393.3 | 9.87 | I | Mᵞ |
| 60 | OsMADS85 | XP_015634793.1 | LOC_Os04g25920 |  | 209 | 23288.9 | 8.54 | I | Mᵞ |
| 61 | OsMADS86 | ABF97222.1 | LOC_Os03g37670 |  | 254 | 28314.9 | 8.51 | I | Mᵞ |
| 62 | OsMADS87 | XP_015629573.1 | LOC_Os03g38610 |  | 249 | 27536.8 | 9.53 | I | Mᵞ |
| 63 | OsMADS88 | XP_025878329.1 | LOC_Os01g18420 |  | 240 | 26415.3 | 10.35 | I | Mᵞ |
| 64 | OsMADS89 | XP_025878343.1 | LOC_Os01g18440 |  | 306 | 33213.0 | 6.25 | I | Mᵞ |
| 65 | OsMADS90 | BAC82950.1 | LOC_Os07g04170 |  | 358 | 39680.0 | 4.74 | I | Mᵝ |
| 66 | OsMADS91 | XP_015636878.1 | LOC_Os01g11510 |  | 604 | 65534.7 | 4.46 | I | Mᵝ |
| 67 | OsMADS92 | BAD53137.1 | LOC_Os01g23750 |  | 406 | 42527.8 | 4.35 | I | Mᵝ |
| 68 | OsMADS93 | BAB67991.1 | LOC_Os01g23760 |  | 407 | 42728.2 | 4.40 | I | Mᵝ |
| 69 | OsMADS94 | BAS71969.1 | LOC_Os01g23770 |  | 323 | 26889.9 | 6.49 | I | Mᵝ |
| 70 | OsMADS95 | BAD53198.1 | LOC_Os01g23780 |  | 406 | 44464.7 | 10.33 | I | Mᵝ |
| 71 | OsMADS96 | BAB92558.1 | LOC_Os01g67890 |  | 483 | 52837.3 | 6.46 | I | Mᵝ |
| 72 | OsMADS97 | BAS75846.1 | LOC_Os01g06842 |  | 277 | 29273.6 | 4.48 | I | Mᵝ |
| 73 | OsMADS98 | XP_015640115.2 | LOC_Os01g68560 |  | 504 | 54129.0 | 4.41 | I | Mᵝ |
| 74 | OsMADS99 | XP_015634794.1 | LOC_Os04g25930 |  | 186 | 20893.4 | 9.98 | I | Mᵞ |

**Note:** OsMADS60 (LOC_Os02g01360) originally identified in rice (Rita *et al*, 2007) is not MADS box gene.
