# Supplementary material for: Asymmetric birth and death of type I and type II MADS-box gene subfamilies in the rubber tree facilitating laticifer development
Source: PLoS One. 2019 Apr 1;14(4):e0214335. doi: 10.1371/journal.pone.0214335 (PMC6443149; doi:10.1371/journal.pone.0214335)
Supplement: S6 Table — (DOCX) [file pone.0214335.s008.docx]

**S6_Table.** MIKC*-type MADS-box reference sequences from other species used in this research.

| **Gene name** | **Species** | **accession** | **Length(aa)** | Type |
| --- | --- | --- | --- | --- |
| MpMADS1 | *Marchantia polymorpha* | GQ334454 | 426 | MIKC* |
| FhMADS1 | *Funaria hygrometrica* | ADB81900.1 | 372 | MIKC* |
| FhMADS2 | *Funaria hygrometrica* | ADB81901.1 | 415 | MIKC* |
| SsMADS1 | *Sphagnum subsecundum* | ADB81896.1 | 364 | MIKC* |
| SmMADS1 | *Selaginella moellendorffii* | XP_002981044.1 | 364 | MIKC* |
| SmMADS2 | *Selaginella moellendorffii* | XP_024539268.1 | 230 | MIKC* |
| EcMADS1 | *Eschscholzia californica* | CAX16990.1 | 361 | MIKC* |
| EcMADS2 | *Eschscholzia californica* | CAX16992.1 | 348 | MIKC* |
| PtMADS1 | *Populus trichocarpa* | XP_024462481.1 | 356 | MIKC* |
| PtMADS2 | *Populus trichocarpa* | XP_024451014.1 | 356 | MIKC* |
| PtMADS3 | *Populus trichocarpa* | XP_024460612.1 | 350 | MIKC* |
| ZmAGL66 | *Zea mays* | NP_001152372.1 | 369 | MIKC* |
| SbMADS1 | *Sorghum bicolor* | XP_021305672.1 | 375 | MIKC* |
| SbMADS2 | *Sorghum bicolor* | XP_021317428.1 | 379 | MIKC* |
| ZmMADS1 | *Zea mays* | AQK83285.1 | 366 | MIKC* |
| HvMADS1 | *Hordeum vulgare* | BAK04308.1 | 387 | MIKC* |
| BdMADS7 | *Brachypodium distachyon* | NP_001288313.1 | 372 | MIKC* |
